# Steroid Biomarkers Revisited – Improved Source Identification of Faecal Remains in Archaeological Soil Material

**DOI:** 10.1371/journal.pone.0164882

**Published:** 2017-01-06

**Authors:** Katharina Prost, Jago Jonathan Birk, Eva Lehndorff, Renate Gerlach, Wulf Amelung

**Affiliations:** 1 Institute of Crop Science and Resource Conservation (INRES) – Soil Science and Soil Ecology, University of Bonn, Bonn, Germany; 2 Institute for Geography - Soil Science, Johannes Gutenberg-University Mainz, Mainz, Germany; 3 Archaeological Heritage Management Rhineland (LVR-Amt für Bodendenkmalpflege im Rheinland), Bonn, Germany; Universidade do Algarve, PORTUGAL

## Abstract

Steroids are used as faecal markers in environmental and in archaeological studies, because they provide insights into ancient agricultural practices and the former presence of animals. Up to now, steroid analyses could only identify and distinguish between herbivore, pig, and human faecal matter and their residues in soils and sediments. We hypothesized that a finer differentiation between faeces of different livestock animals could be achieved when the analyses of several steroids is combined (Δ^5^-sterols, 5α-stanols, 5β-stanols, epi-5β-stanols, stanones, and bile acids). We therefore reviewed the existing literature on various faecal steroids from livestock and humans and analysed faeces from old livestock breed (cattle, horse, donkey, sheep, goat, goose, and pig) and humans. Additionally, we performed steroid analyses on soil material of four different archaeological periods (sites located in the Lower Rhine Basin, Western Germany, dating to the Linearbandkeramik, Urnfield Period / Bronze Age, Iron Age, Roman Age) with known or supposed faecal inputs. By means of already established and newly applied steroid ratios of the analysed faeces together with results from the literature, all considered livestock faeces, except sheep and cattle, could be distinguished on the basis of their steroid signatures. Most remarkably was the identification of horse faeces (via the ratio: epi-5β-stigmastanol: 5β-stigmastanol + epicoprostanol: coprostanol; together with the presence of chenodeoxycholic acid) and a successful differentiation between goat (with chenodeoxycholic acid) and sheep/cattle faeces (without chenodeoxycholic acid). The steroid analysis of archaeological soil material confirmed the supposed faecal inputs, even if these inputs had occurred several thousand years ago.

## Introduction

Archaeological excavations frequently discover organic-rich topsoil material potentially influenced by human or animal remains, but not necessarily mixed with archaeological artefacts. This buried former topsoil material can be preserved in trenches, pits, post-holes or wells [1–2] while still carrying information on the human impact in its chemical signature. Molecular markers may provide indications for agricultural practices outside settlements (off-site features; [2–7]) or for specific husbandry systems, fireplaces, gardens, middens, latrines, and sewage channels inside settlements (on-side features; [8–11]). Among the manifold methods used in archaeology and related disciplines for detection and source identification of faecal matter [12–13], steroid analysis is one promising tool if the archaeological context is indicating a faecal input, but macroscopic evidence lacking. This is due to the fact that under oxygen-deficient conditions, steroids can be well preserved and serve as biomarkers for a faecal input that occurred hundreds to thousands of years ago [4, 6, 9, 14–15]. Furthermore, steroids (in particular 5β-stanols) show a low water solubility and are mainly adsorbed to particulate organic matter. As a consequence they are not prone to leaching but bind to the soil matrix [16–17].

**Fig 1:**
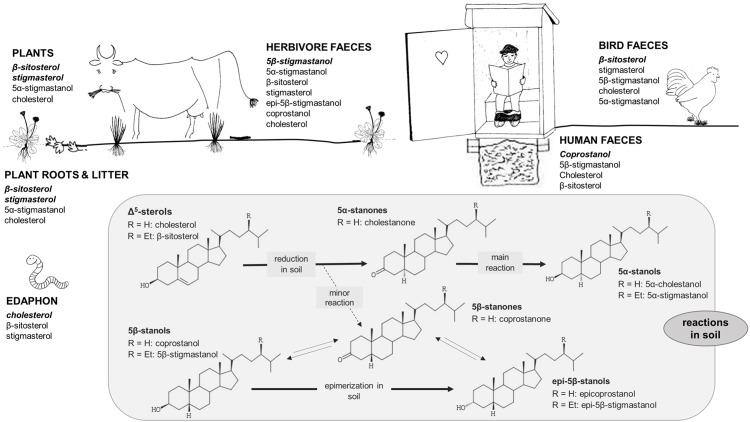
Δ^5^-sterols, stanols and stanones in the environment as compiled from literature data. Dominating and characteristic steroids are written in bold italics. Reactions in soil modified from Bull et al. [27]. Literature data compilation from [19–24, 26–36,38–44].

Steroids occur in the environment in plants, fungi and in animal (including human) tissues and faecal remains, with stanols being the steroids that are most often used as biomarkers in environmental and archaeological studies (Fig 1).

Stigmasterol and β-sitosterol are the typical Δ^5^-sterols for plant biomass, whereas cholesterol is the dominating Δ^5^-sterol in most animal tissues [18]. However, cholesterol is also a component of nearly all eukaryotic cells [19] and can be found in animals (e.g. the soil meso- and macrofauna), plants (0–70% of total sterols), root exudates, as well as in several fungal species [20–25]. Thus, cholesterol as well as stigmasterol and β-sitosterol can be widespread in soil (Fig 1). Stanols are mostly produced by microbial processes from Δ^5^-sterols. The 5α-stanols, 5α-stigmastanol and 5α-cholestanol, are produced in the course of microbial degradation processes from their sterol precursors, i.e. β-sitosterol and cholesterol, in the environment ([26–28]; Fig 1). However, small amounts of 5α-stanols have also been found in fresh plant and animal tissue [29–31]. In contrast, 5β-stanols and epi-5β-stanols are mainly produced by specialized microorganisms in the gut of higher animals, but only to a lesser extent in the environment ([27,32–36]; Fig 1). Stanones have rarely been analysed yet [2,8,37]. They are intermediates that are formed in the course of the transformation of Δ^5^-sterols to 5β-stanols, 5α-stanols, and epi-5β-stanols, both in the gut of higher animals as well as in the environment ([27,32–33]; Fig 1). Hence, Δ^5^-sterols, stanols, and stanones reach the soil by different pathways, e.g., via dead plant or animal material, via root exudates, faeces, or soil (micro-) flora and fauna, or they are directly formed in soil by microorganisms from precursor sterols (Fig 1).

Vertebrate faeces from different species show remarkable differences in their contents and distribution of particular steroids (e.g. Δ^5^-sterols, stanols, stanones, and bile acids), due to differences in the diet (herbivore, omnivore, and carnivore), in the ability to produce endogenous steroids, and due to the presence or absence of different anaerobic bacteria in the digestive systems [13,38]. It is thus that steroids can be used for the identification of faeces and faecal inputs into soils and sediments [27]. Mostly 5β-stanols have been used for this purpose, with human faeces containing large amounts of coprostanol, while those of herbivores show larger 5β-stigmastanol compared to coprostanol contents [38–42]. For bird and dog faeces only small contents of 5β-stanols have been observed; here, Δ^5^-sterols dominate steroid profiles [38,43–44]. In general, however, the ubiquitous occurrence of Δ^5^-sterols, as well as their transformation to stanols in the environment, make their use as specific faecal biomarkers difficult. In contrast, 5β-stanols, 5β-stanones, and epi-5β-stanols should be—due to their main production in the gut of higher animals—more specific, although 5β-stanols have also been found in small background concentrations in soils that have not been fertilized with faeces, especially in anaerobic environments [33,36]. Detecting different Δ^5^-sterols, stanols, epi-5β-stanols and stanones together in one analysis should thus be a promising approach, revealing a more complex steroid profile and thereby providing deeper insights into the source identification of different faecal residues than achieved by the analyses of one steroid class alone [27,45].

To account for the potential of using the steroid composition for source assignment, several steroid ratios for detection of faecal input into soils and sediments have been proposed to date. These ratios usually use 5β-stanols as well as their transformation products (Fig 1). The most widely used ratio for a general detection of a faecal input is that from Grimalt and co-authors [46], originally applied for detection of human derived faecal matter from sewage in sediments. It relates the human 5β-stanol coprostanol to the sum of coprostanol and the cholesterol transformation product 5α-cholestanol (with a threshold value of >0.7 indicating a faecal input). To account for microbial degradation processes that are leading to transformation of coprostanol to epicoprostanol, Bull et al. [47] expanded this ratio by adding epicoprostanol to the numerator and denominator (using again a threshold value of >0.7 as an indication for a faecal input). Several studies [27,47–48] pointed out that it is also essential to consider the steroid composition from soils nearby that had not received any faecal input (control samples) in order to be able to trace faecal inputs even when certain threshold values for steroid ratios fail to indicate so (e.g. by comparing steroid ratios of the soils with those of the control [4]).

**Fig 2:**
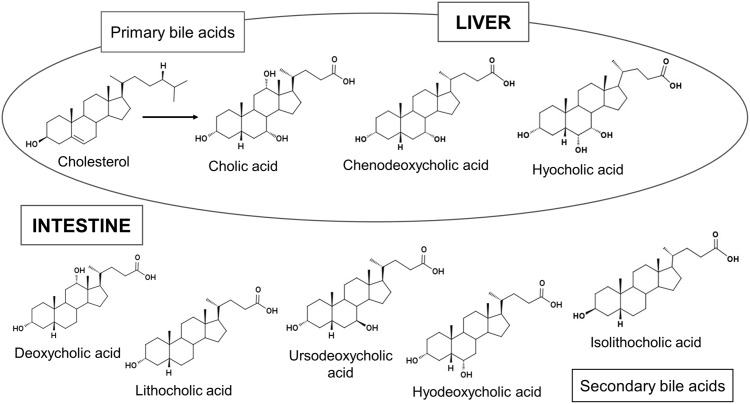
Primary and secondary bile acids (modified after Bull et al., [27]).

The commonly used steroid ratios still do not take advantage of the full potential of the steroid spectrum, because they do not consider bile acids. Bile acids are likely the most specific markers for a faecal input, due to their exclusive occurrence in vertebrate faeces [49–50]. Furthermore, bile acids are more resistant to degradation than Δ^5^-sterols, stanols, and stanones [51] and can therefore still reveal an ancient faecal input into soils where other markers have already been degraded [2, 9]. The primary bile acids (cholic acid and chenodeoxycholic acid in humans and chenodeoxycholic acid and hyocholic acid in pigs) are formed in the liver from cholesterol, are excreted into the intestine and then transformed microbially to secondary bile acids (Fig 2; [27,52]). In the human body cholic acid, chenodeoxycholic acid, and deoxycholic acid are returned to the liver, whereas most of the secondary bile acid lithocholic acid is excreted in faeces [53]. Due to different bile acid composition and metabolism, bile acid profiles of vertebrates (including humans) may differ significantly [27,53–54]. Nevertheless, there has been no study yet that combined Δ^5^-sterols, 5α-stanols, 5β-stanols, epi-5β-stanols, stanones, and bile acids for a differentiation between different livestock faeces (S1 Table). Additionally, factors probably influencing the steroid pattern of faeces, like the utilized fodder (e.g. grass, silage, concentrates) or the extent of animal breeding from their first domestication onwards, have so far rarely been considered ([42]; S2 Table).

In summary, current faecal source assignment using chemical biomarker analyses mainly distinguished between human, porcine and herbivore faecal matter [27], as well as between ruminant and non-ruminant sources (by the presence of archaeol, a archaeal dialkyl glycerol ether; [41]). We are not aware of any study that succeeded so far in differentiating between faeces from different herbivores. It has to be considered, however, that applying such methods to recent faeces does not necessarily apply to faeces of an archaeological context, due to different fodder used in earlier times than used nowadays, and due to remaining uncertainties on the preservation conditions of steroid composition in the course of archaeologically relevant time scales. The main objective of this work, was, therefore, to improve source identification for common livestock faeces by their steroid composition (considering also the impact of diet and breed) and to elucidate if the identified steroid compositions can also be detected in respective archaeological soil material. To achieve this aim, we combined for the first time the assessment of Δ^5^-sterols, 5α-stanols, 5β-stanols, epi-5β-stanols, stanones, and bile acids for a differentiation between different livestock faeces, including faeces from different herbivore livestock, and we complemented our data set by a compilation of literature data. To account for a different feeding management of livestock in modern and ancient agriculture, our study is also the first one that provides steroid composition data of faeces from old livestock breed that had exclusively been fed with traditional fodder. We did not consider faeces from livestock that were fed with silage or concentrate to ensure comparability of results from steroid analysis in faeces and in archaeological soil material and chose archaeological soil samples with strong indications on ancient livestock management or a human faecal input.

## Materials and Methods

### Literature compilation

We compiled studies on faecal steroids of humans and livestock. We provide information on the animals and their diet (S2 Table), as well as the suite of analysed steroids and the method of quantification (S1 Table). For discussion we considered only those studies that reported quantified steroid contents (see S7–S9 Tables). We did not consider campesterol and its transformation products (5α-campestanol, 5β-campestanol, and epi-5β-campestanol), because they have not been used for steroid ratios, yet, and because the transformation products were commercially not available (or not affordable).

### Faecal samples

Faeces from old livestock breed (cattle, horses, donkey, sheep, goats, geese, and pigs) were collected from the fields and enclosures of different institutions, farms and private breeders without affecting the animals (for more detailed information see S3 Table). All institutions and breeders providing faecal material confirmed that the animals had been fed exclusively with fodder that has been used in agriculture hundreds to thousands of years ago, i.e. without concentrates and silage. However, in order to elucidate the influence of silage feeding, also faeces from cows that had been fed with grass and red clover silage were included in the sample design (data presented in S7–S9 Tables). Additionally, two women and one man following a vegetarian or a diet with rare fish and meat consumption provided faecal samples and gave their written consent to use them for this study. All faecal samples were taken as fresh samples, cooled during transport, and subsequently frozen. After freeze drying all samples from each species were milled, mixed and analysed as composite samples. This allowed us to present a realistic spectrum of steroid contents and a realistic range of analytical precision. In order to account for possible variations of the signals across different animals, we compared and compiled our own data with literature data, which allowed us to provide the most realistic variation of steroid ratios in faeces that can likely be achieved to date. We are aware that in theory this includes the risk of a bias when comparing results from flame-ionization detection and mass-selective detection, since the former procedure is not able to identify co-eluting peaks, while the latter may be more sensitive to matrix-selective ionization reactions. Hence, all comparisons have to rely on the assumption that all methods have been robustly tested regarding these issues.

### Study sites and soil samples

All archaeological soil samples were taken from archaeological sites located in the Lower Rhine Basin, Western Germany (Table 1; [55–57]).

**Table 1:** Age, basic characteristics and archaeological sampling sites of the soil samples.

Site (geographical coordinates)	Sample	Age^†^	pH^‡^	C_org_ (g kg^-1^)	N_t_(g kg^-1^)
**Dormagen** (51° 5’ 36.31” N, 6° 50’ 20.59” E)	Cesspit	Roman Age (1^st^-4^th^ century AD)	7.2	3.0	0.38
	Brown stable drain filling		7.1	9.0	0.76
	Green stable drain filling		7.1	3.1	0.40
	Stable area		7.0	3.1	0.45
	Control		7.0	1.8	0.32
**Inden** (50° 51’ 45.83” N, 6° 21’ 25.52” E)	Sewer ditch (70 cm depth)	Roman Age (c. 0–450 AD)	x	1.8	0.05
	Sewer ditch (80 cm depth)		x	10.6	0.13
	Control		6.7	ND	0.05
**Düren-Arnoldsweiler** (50° 51‘ 3” N, 6° 30‘ 25” E)	Well with box-shaped wooden lining	Linearbandkeramik (c. 5300–5000 BC)	7.5	2.0	0.10
	Tree trunk well	Bronze age (c. 1440 BC) / Urnfield Period (c. 1200–700 BC)	6.6	1.3	0.11
	Water hole with wickerwork revetment	Early to Middle Iron Age (544–389 BC)^§^	6.0	1.5	0.13

^†^ timescale following Meurers-Balke et al., 1999;

^‡^ in 0.01 M CaCl_2_

^§^ dating according to Husmann and Cziesla, 2014 and Jürgens, 2014

**Fig 3:**
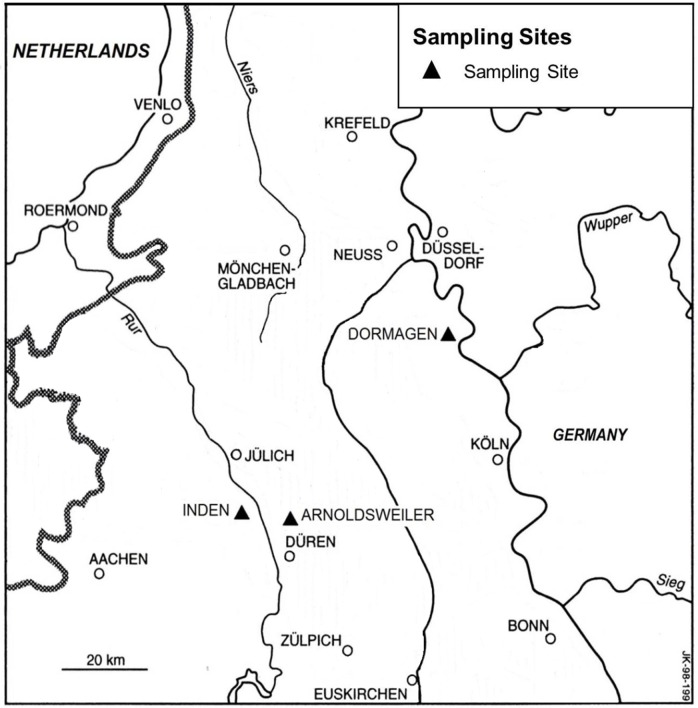
Location of excavation sites in the loess region of western Germany; for sample description and photos see S1–S5 Figs.

The necessary permits were obtained from the Archaeological Heritage Management Rhineland, which complied with all relevant regulations. Here, three different sites were chosen, a supposed latrine and a stable from a Roman fort in Dormagen, a sewer ditch from a Roman “Villa Rustica” in Inden, as well as two wells and one water hole in Düren-Arnoldsweiler originating from different archaeological periods (Fig 3).

The sample set of the site “Dormagen” contained two samples of a horse stable drain (“brown stable drain filling” and “green stable drain filling”; S1–S3 Figs), one sample from the horse stable area in close proximity to the stable drain (“stable area”; S1 and S3 Figs), one sample from a supposed cesspit (“cesspit”; S1 and S3 Figs), and one control sample from an area of a combination building outside the stable (S3 Fig, all sampling points are marked). The former surface ground could not be reconstructed anymore as there had been several construction activities ensuing the placing and removal of construction waste. It is however known that the samples had been covered by a 30–160 cm thick layer of construction waste.

The sample set of a sewer ditch from a Roman “Villa Rustica” in Inden contained three samples, two from inside the ditch (in 70 and 80 cm depth) and one control sample from outside but in close proximity to the ditch (100 cm depth; S4 Fig).

At the site Düren-Arnoldsweiler three different samples had been taken. One sample from the fillings of a well with box-shaped wooden lining dating to the Linearbandkeramik (Linear Pottery culture, LBK, 5098 ± 5 BC), one from a tree trunk well from the Bronze Age / Urnfield Period (1440–700 BC) and one from a water hole with wickerwork revetment from the Early to Middle Iron Age (Hallstatt D Period / Latène A Period, 544–389 BC; S5 Fig [56–58]). According to pollen analyses, the LBK and the Iron Age well had been situated in a settlement, whereas the Bronze Age well was located on a pasture [57,59]. Due to their location under the permanent groundwater table all constituents and fillings of the wells were well preserved [56–57].

Further information about the archaeological samples and the archaeological and historical context is presented in S1–S3 Texts.

### Basic soil characteristics

After sampling the soil was air-dried. For further analyses the soil was freeze dried, sieved to <2 mm and sub-samples were milled for total carbon, total nitrogen and steroid analyses. Total carbon and nitrogen contents were determined after dry combustion [60] with an elemental analyzer (Fisons NA 2000). Carbonate content was measured with the Scheibler method [61]. Soil organic carbon (C_org_) was calculated from total carbon and carbonate carbon. The pH-value was determined in 0.01 M CaCl_2_ using a soil to solution ratio of 1:2.5 ([62]; Table 1).

### Steroid analysis

All solvents used were of HPLC-grade and acids were pro analysis grade. Water was purified using a Millipore Synergy water treatment system (Schwalbach, Germany).

#### Steroid extraction, separation and derivatization

Steroid analyses of faeces and soils followed the protocol of Birk et al. [45] with modifications concerning sample extraction and quantification. For soil analyses, in brief, 10 g of dried and milled soil (four replicates) was subsequently extracted with dichloromethane/methanol (2:1, v/v) and dichloromethane/methanol (1:3, v/v) using accelerated solvent extraction (ASE Dionex 350; at 100°C, 5 min heating time, 5 min static time and 3 cycles). Before the extraction of the soil three replicates were spiked with recovery (IS 1) standards (5β-pregnan-3α-ol-20-one, 5β-pregnan-3α-ol, and isodeoxycholic acid), one was extracted without spiked recovery standards (= matrix sample). After extraction the total lipid extracts were evaporated (Büchi, Rotavapor R-210/R-215) and dried under a gentle stream of nitrogen. Afterwards the dried extracts were saponified by adding 3.5 mL 5% KOH in methanol. Reaction was allowed over night (10–14 h) at room temperature. Afterwards, the saponified extracts were separated into a neutral fraction (including Δ^5^-sterols, stanols and stanones) and an acidic fraction (including the bile acids). For this purpose extracts were transferred into separatory funnels, 10 mL Millipore water was added, and a repeated liquid-liquid extraction with chloroform (3x15 mL) performed. In the end the neutral fraction was released, the remaining solution acidified with 1 M HCl (to a pH ≤ 2), and a further liquid-liquid extraction with chloroform (3x15 mL) performed.

Neutral fraction: After drying of the eluates and a re-dissolving in hexane, the neutral fraction was fractionated by solid phase extraction (SPE) using 5% deactivated silica gel (Merck Grade 7734, pore size 60Ǻ, 70–230 mesh) and (i) 5 mL hexane (for preconditioning), (ii) 5 mL hexane, (iii) 3 mL dichloromethane and (iv) 2 mL dichloromethane/acetone (2:1, v/v). The second fraction was discarded; the third and fourth fraction (containing the Δ^5^-sterols, stanols and stanones) were combined and dried under a gentle stream of nitrogen. Finally, Δ^5^-sterol, stanol, and stanone extracts were silylated by adding the derivatization agent, i.e. 22.9% 1,1,3,3,3-Hexamethyldisilazane (HMDS) and 7.7%, Trimethylchlorosilane (TMCS) dissolved in 69.4% Pyridine (w/v) (Sylon HTP; Sigma Aldrich), and heating the mixture at 70°C for 1 h.

Acidic fraction: The acidic fraction was methylated by re-dissolving the dried eluates in 1 mL 1.25 M HCl in methanol (Sigma Aldrich) and heating at 80°C for 2 h. The methyl esters were extracted (after adding 1 mL Millipore water) by repeated liquid-liquid-extraction with 3x1 mL hexane. For a separation into a methylated fatty acid and a methylated bile acid fraction by SPE activated silica gel (Merck Grade 7734, pore size 60Ǻ, 70–230 mesh) was preconditioned with (i) 5 mL hexane/dichloromethane (2:1, v/v). The methylated acidic fraction (in hexane) was transferred onto the column and eluted with (ii) 4 mL dichloromethane/hexane (2:1, v/v) and (iii) 5 mL dichloromethane/methanol (2:1, v/v). The second fraction was discarded, the third fraction, containing the bile acid methyl esters, was dried and derivatized by adding 50 μL toluene and N,O-bis(trimethylsilyl)trifluoroacetamide (BSTFA; Sigma Aldrich, Germany) containing N-trimethylsilylimidazole (TSIM; Sigma Aldrich, Germany) (98:2, v/v) and heating at 80°C for 1 h.

The analysis of the faeces was performed similarly to that of soils but with smaller sample weights (50–100 mg) and an additional splitting of the extracts when needed. After splitting of the extracts three replicates were spiked with recovery (IS 1) standards (5β-pregnan-3α-ol-20-one, 5β-pregnan-3α-ol, and isodeoxycholic acid), one was analysed without being spiked (= matrix sample).

#### Steroid measurements and quantification

Both the bile acid methyl ester and the Δ^5^-sterol, stanol, and stanone fraction were spiked with 5α-cholestane (second internal standard) before analysis by gas chromatography-mass spectrometry (GC/MS) with an Agilent 5973 quadrupole mass spectrometer coupled to an Agilent 6890 gas chromatograph (Agilent, Böblingen, Germany).

Gas chromatographic separation of the steroids was carried out with an Optima-5 MS column (30 m x 0.25 mm x 0.25 μm; Macherey-Nagel, Düren, Germany). The injection port was set to 250°C and samples were injected in splitless mode.

For analyses of Δ^5^-sterol and stanol derivates and of stanones the column temperature program was 80°C (held 1.5 min) to 265°C at 12°C min^-1^, to 288°C at 0.6°C min^-1^, to 300°C at 10°C min^-1^, and to 340°C at 25°C min^-1^ (held 5 min). For analyses of bile acid derivated the column temperature program was 80°C (held 1.5 min) to 250°C at 20°C min^-1^, to 287°C at 1.2°C min^-1^ (held 5 min), and to 340°C at 25°C min^-1^ (held 1.5 min). The mass spectrometer was operated in the electron ionization mode at an electron energy of 70 eV and an ion source temperature of 280°C. Scan mode and the comparison with external standards were used to verify peak identity; measurements in selected ion monitoring mode (SIM) were carried out for quantification. S4 Table shows the steroid structures, the retention times, and the selected characteristic ion fragments. S6 to S7 Figs show standard solution chromatograms and S8 to S26 Figs the mass spectra of the analysed steroids. All steroids were quantified by an external standard series (five point calibration curves) for each analyte (for information on suppliers of each steroid see S4 Table; amounts of standards spiked to the standard series are presented in S5 and S6 Tables) and with sample matrix for each sample. To this end, the peak areas of the steroids in the samples and in the external standards, respectively, were divided by the peak area of the second internal standard. Using these ratios of the external standard series, calibration curves for each substance were calculated.

For the soil samples the mean recovery (± standard deviation) of the first internal standards pregnanolone (5β-pregnan-3α-ol-20-one), desoxypregnanolone (5β-pregnan-3α-ol), and isodeoxycholic acid ranged from 88 ± 28%, 82 ± 12%, 52 ± 23%, respectively. Small recoveries for the bile acid were attributed to sorption processes, as recovery standards were spiked to the soil before extraction and as the mean recovery of the bile acid in the faecal samples was distinctly larger. For the faecal samples the mean recovery (± standard deviation) of the first internal standards pregnanolone (5β-pregnan-3α-ol-20-one), desoxypregnanolone (5β-pregnan-3α-ol), and isodeoxycholic acid ranged from and 87 ± 19%, 99 ± 16%, 102 ± 14%, respectively. The limit of quantification (LOQ) was 2 ng g^-1^ soil for all Δ^5^-sterols, stanols, and stanones as well as for isodeoxycholic acid, isolithocholic acid, and lithocholic acid and 5 ng g^-1^ for all other bile acids (the LOQ was determined as signal-to noise ratio of 10:1). We did not use IS 1 recoveries to correct the determined steroid concentrations (because isotope-labelled steroids were not available for each analysed compound).

## Results and Discussion

### Steroid profiles of faeces from old livestock breeds and humans

We present in Tables 2 and 3 steroid contents of the analysed faecal samples of this study and in S7–S9 Tables steroid contents from the literature. Total steroid contents, i.e. the sum of Δ^5^-sterols, stanols, stanones (Table 2) plus the bile acids (Table 3), of faeces from herbivore species (cattle, sheep, goats, horses, donkey, and geese) ranged between 464 μg g^-1^ (geese) and 4932 μg g^-1^ (heck cattle), whereas those of omnivore species comprised larger total contents, ranging from 5480 μg g^-1^ for Mangaliza pig faeces to even 15,116 μg g^-1^ for human faeces (Tables 2 and 3).

**Table 2:** Sterol, stanol and stanone contents of faeces from old livestock breeds and humans.

Steroid (trivial name)	Steroid content (μg g^-1^ dry matter)								
	Heck Cattle^a^	Sheep^b^	Goats^b^	Horses^a^	Donkey^c^	Geese^b^	T.-Pigs^b^	M.-Pigs^b^	Humans^d^
Laboratory replicate	3	3	3	3	3	3	3	3	3
	**Δ**^**5**^**-Sterols**								
Cholesterol	319 ± 72	302 ± 35	252 ± 3	69 ± 6	30 ± 6	72 ± 15	125 ± 20	113 ± 33	759 ± 83
Stigmasterol	64 ± 1	65 ± 12	18 ± 4	53 ± 6	35 ± 1	11 ± 3	12 ± 1	16 ± 1	51 ± 0
β-Sitosterol	272 ± 72	497 ± 116	582 ± 99	575 ± 63	208 ± 47	155 ± 48	157 ± 13	209 ± 70	313 ± 53
	**5β-Stanols**								
Coprostanol	251 ± 17	524 ± 63	63 ± 7	82 ± 4	92 ± 5	15 ± 3	772 ± 93	976 ± 129	6940 ± 140
5β-Stigmastanol	2440 ± 665	3223 ± 834	914 ± 95	1025 ± 134	688 ± 7.5	80 ± 12	1701 ± 188	1799 ± 418	3168 ± 333
	**Epi-5β-stanols**								
Epicoprostanol	33 ± 2	27 ± 6	13 ± 2	128 ± 4	21 ± 1	2 ± 1	30 ± 2	62 ± 11	87 ± 11
Epi-5β-stigmastanol	215 ± 35	182 ± 36	227 ± 13	826 ± 90	46 ± 3	6 ± 1	37 ± 6	54 ± 19	0 ± 0
	**5α-Stanols**								
5α-Cholestanol	90 ± 1	157 ± 26	62 ± 6	41 ± 4	32 ± 12	20 ± 5	103 ± 15	100 ± 22	96 ± 6
5α-Stigmastanol	553 ± 141	1387 ± 425	328 ± 3	208 ± 26	170 ± 21	65 ± 20	179 ± 23	423 ± 126	164 ± 29
	**Stanones**								
Coprostanone	154 ± 36	143 ± 25	21 ± 6	23 ± 5	16 ± 5	5 ± 1	45 ± 4	37 ± 8	111 ± 7
Cholestanone	109 ± 24	156 ± 36	27 ± 10	3 ± 3	9 ± 3	2 ± 1	62 ± 4	126 ± 30	453 ± 27
**Others**									
4-Cholesten-3-one	0 ± 0	0 ± 0	0 ± 0	0 ± 0	0 ± 0	0 ± 0	0 ± 0	0 ± 0	0 ± 0
6-Ketocholestanol	0 ± 0	0 ± 0	0 ± 0	0 ± 0	0 ± 0	0 ± 0	0 ± 0	0 ± 0	0 ± 0
∑ steroids^e^	4501	6662	2508	3034	1346	433	3221	3916	12141

All values are means ± standard deviation

^a^ composite sample of n = 5 faeces samples of different individuals,

^b^ composite sample of n = 10 faeces samples of different individuals;

^c^ faeces sample of n = 1 individual,

^d^ composite sample of n = 3 faeces samples of different individuals, T.-pigs = Turopolje pigs, M.-pigs = Mangaliza pigs

^e^ sum of sterols, stanols, and stanones

**Table 3:** Bile acid contents and bile acid ratios of faeces from old livestock breeds and humans.

Steroid (trivial name)	Bile acid contents (μg g^-1^ dry matter)								
	Heck cattle^†^	sheep^‡^	goats^‡^	horses^†^	donkey^§^	geese^†^	T.-pigs^†^	M.-pigs^†^	humans^†^
Laboratory replicate	3	3	3	3	3	3	3	3	3
	**Bile acids**								
IDCA (isodeoxycholic acid)^¶^	5.0	9.6	3.4	10.5	8.3	0 ± 0	6.8 ± 0	0 ± 0	0 ± 0
ILCA (isolithocholic acid)	9.7 ± 3.2	0.3 ± 0.1	0.8 ± 0.1	0.5 ± 0.1	5.7 ± 0.0	0.1 ± 0.0	60 ± 1.6	131 ± 13	307 ± 41
LCA (lithocholic acid)	40 ± 18	5.0 ± 0.9	5.8 ± 0.0	15 ± 2.2	34 ± 1.4	5.3 ± 0.4	224 ± 15	334 ± 34	562 ± 40
DCA (deoxycholic acid)	376 ± 90	48 ± 1.7	198 ± 81	39 ± 3.8	41 ± 0.9	1.4 ± 0.6	26 ± 3.7	5.3 ± 2.2	2088 ± 262
CDCA (chenodeoxycholic acid)	0 ± 0	0 ± 0	5.7 ± 0.5	42 ± 2.9	0 ± 0	24 ± 1.9	0 ± 0	0 ± 0	18 ± 4.5
HDCA (hyodeoxycholic acid)	0 ± 0	0 ± 0	0 ± 0	0 ± 0	0 ± 0	0 ± 0	1996 ± 157	1006 ± 333	0 ± 0
UDCA (ursodeoxycholic acid)	0 ± 0	0 ± 0	0 ± 0	0 ± 0	0 ± 0	0 ± 0	254 ± 60	87 ± 29	0 ± 0
∑ bile acids	431	63	213	107	89	31	2566	1564	2975
	**Bile acid ratios**								
DCA / LCA	9 (5–21)	10 (8–12)	34 (20–48)	2.6 (2.1–3.4)	1.2 (1.1–1.3)	0.3 (0.2–0.4)	0.1 (0.09–0.14)	0.02 (0.01–0.02)	3.7 (3.0–4.5)
DCA / CDCA	-	-	35 (19–53)	0.9 (0.8–1.1)	-	0.06 (0.03–0.09)	-	-	114 (80–171)
CDCA / LCA	-	-	1.0 (0.9–1.1)	2.8 (2.3–3.5)	-	4.5 (3.9–5.4)	-	-	0.03 (0.02–0.04)
HDCA/LCA	-	-	-	-	-	-	9 (8–10)	3.0 (1.8–4.5)	-

All values are means ± standard deviation

^†^ composite sample of n = 5 faeces samples of different individuals,

^‡^ composite sample of n = 10 faeces samples of different individuals,

^§^ faeces sample of n = 1 individual, T.-pigs = Turopolje pigs, M.-pigs = Mangaliza pigs,

^¶^ recovery standard (quantified by the method of standard addition, n = 1)

Total and single steroid contents, which are reported in the literature, vary considerably (S7–S9 Tables). One reason for this observation is the fact that there is up to now no standard methodology for steroid analysis making a comparison and a correlation of results from different studies very difficult [27]. However, also here a trend can be observed of large total steroid contents in human faeces, smaller contents in pig and smallest contents in herbivore faeces (S7–S9 Tables; [38;54]).

The predominating compounds in all analysed faeces of our study were either 5β-stigmastanol or coprostanol (both commonly used as faecal markers; Table 2). The faeces of herbivores contained largest contents of 5β-stigmastanol, followed either by 5α-stigmastanol or by β-sitosterol, except for the horse faeces, which exhibited maximum contents of 5β-stigmastanol and epi-5β-stigmastanol (Table 2). The only exception to the observed predominance of 5β-stigmastanol in herbivore faeces was the steroid profile of the goose faeces with the plant sterol β-sitosterol showing largest contents (followed by 5β-stigmastanol). The sum of phytosterol contents in herbivore faeces (stigmasterol, and β-sitosterol) and their transformation products (5β-stigmastanol, epi-5β-stigmastanol, and 5α-stigmastanol) comprised 64–89% of the total contents of Δ^5^-sterols, stanols and stanones, reflecting the plant dominated diet of the animals.

We could not detect 4-cholesten-3-one or 6-ketocholestanol in any of the analysed faeces (Table 2). Both compounds are produced in the course of cholesterol transformation [16, 27,63], but, to our knowledge, so far none of them have ever been analysed in studies on faecal samples.

These results for the herbivores corresponded to those presented in a study Gill et al. [41] with 5β-stigmastanol, 5α-stigmastanol and β-sitosterol dominating the steroid spectra of sheep and cow faeces (for cows fed with grass and hay) and 5β-stigmastanol, epi-5β-stigmastanol, and β-sitosterol dominating the steroid spectra of horse faeces (S7 Table). Concerning only the two faecal markers, all herbivore faeces comprised in our study (with five to 15 times) markedly larger contents of 5β-stigmastanol compared to coprostanol, confirming the use of 5β-stigmastanol as biomarker for herbivore faeces (Table 2; [48]). In contrast, results of a study from Shah and co-authors [64] showed significantly smaller 5β-stigmastanol contents for all animals compared with the results of our and of all other studies (Table 2; S7 and S8 Tables), we therefore excluded results from Shah et al. [64] from the further discussion.

Intriguingly, and in contrast to Bull et al. [27], we observed a predominance of 5β-stigmastanol over coprostanol in pig faeces (two times larger 5β-stigmastanol contents). In fact, studies that analysed pig faeces have not been consistent in this regard, as one reported on equally large contents [38], whereas another one observed slightly larger 5β-stigmastanol than coprostanol contents ([42]; S7 Table). In our study the diet of both pig breeds had consisted dominantly of fruits and vegetables (S3 Table). It is therefore very likely that this diet led to the large proportion (64–65%) of plant sterols (β-sitosterol and stigmasterol) and their transformation products (5β-stigmastanol, epi-5β-stigmastanol, and 5α-stigmastanol) relative to total Δ^5^-sterol, stanol, and stanone contents (Table 2) in the pigs’ faeces. The results suggest that the exact 5β-stanol (faecal marker) pattern of animal faeces is more strongly affected by the actual diet than hitherto assumed.

Coprostanol comprised the largest steroid contents in human faeces (Table 2), comprising alone 57% of all analysed steroids. This finding is in accordance to earlier studies on human faeces ([38–39]; S7 Table). We thus confirm that the large predominance of coprostanol is characteristic for human faeces. Although coprostanol is also one dominating steroid in pig faeces, it is no distinct marker for omnivore faeces in general. Due to the observed influence of the actual diet on faecal 5β-stanol contents, it is thus important to include other faecal markers in steroid analyses, which are less dependent on the diet, like e.g. bile acids.

The analysis of bile acids revealed for the faeces of humans and ruminants (i.e. cows, goats, and sheep) a predominance of deoxycholic acid (DCA), making up 70% to 93% of total bile acid contents (Table 3). Chenodeoxycholic acid (CDCA) could only be detected in the faeces of horses, geese, goats, and humans, whereas hyodeoxycholic (HDCA) and ursodeoxycholic acid (UDCA) occurred exclusively in pig faeces, with HDCA being their predominating bile acid (Table 3). Lithocholic acid (LCA) was present in all analysed faeces, but the largest absolute contents were found in the faeces of humans and pigs (but also large relative contents in donkey faeces). These results are comparable to those in the literature on faecal bile acids, with DCA being the predominating bile acid of ruminant and human faeces, HDCA occurring exclusively in pig faeces, and CDCA being present in horse and human faeces ([27,40,54,65]; S9 Table). However, in contrast to our results, Tyagi et al. [54] also detected CDCA in pig faeces (S9 Table).

### Detection and source identification of faecal matter

Steroid biomarkers have been used in the literature for both a detection of faecal inputs into the environment (discussed in this section), but also for a source assignment of the faecal input (discussed in the next section). For both purposes, several steroid ratios have been established so far, using Δ^5^-sterols, stanols, stanones, and bile acids [38,42,46–47,54,66].

#### Markers for detection of faeces in the environment

Commonly applied steroid ratios for detection of faecal material in the environment, i.e. showing only the presence of faecal material without any source assignment, have been established on human faeces, but have not been tested on faeces from livestock animals, yet. In order to fill this gap and to gain knowledge on the applicability of commonly used ratios for different kind of faeces, we tested whether the established ratios perform well on faeces from different livestock animals and humans. We only applied ratios using 5β-stanols and 5β-stanones and their degradation products (epi-5β-stanols) in relation to 5α-stanols and 5α-stanones [46–47] on our results and on those from the literature (Table 4; S7 and S8 Tables), but no ratios using Δ^5^-sterols, as these steroids are not causally linked to the presence of faecal material (Fig 1).

**Table 4:** Steroid ratios for detection and for source identification of faecal matter applied on faecal samples (old livestock breed and humans).

No.	Ratio	Heck cattle	Sheep	Goats	Horses	Donkey	Geese	Pigs	Humans
**Ratios for mere detection of faecal matter**									
I	(coprostanol + epicoprostanol) / (5α-cholestanol + coprostanol + epicoprostanol)^†^	0.76 **✔**	0.78 **✔**	0.55 **+/-**	0.84 **✔**	0.78 **✔**	0.46 **+/-**	0.90 **✔**	0.99 **✔**
II	(5β-stigmastanol + epi-5β-stigmastanol) / (5α-stigmastanol + 5β-stigmastanol + epi-5β-stigmastanol) ^‡^	0.83 **✔**	0.71 **✔**	0.78 **✔**	0.90 **✔**	0.81 **✔**	0.57 **+/-**	0.86 **✔**	0.95 **✔**
III	coprostanol / (5α-cholestanol + coprostanol) ^§^	0.74 **✔**	0.77 **✔**	0.50 **+/-**	0.67 **+/-**	0.74 **✔**	0.43 **+/-**	0.90 **✔**	0.99 **✔**
IV	coprostanone / (cholestanone + coprostanone) ^§^	0.59 **+/-**	0.48 **+/-**	0.44 **+/-**	0.88 **✔**	0.65 **+/-**	0.70 **✔**	0.30 **+/-**	0.20 **✘**
**Ratios for source identification of faecal matter**									
V	coprostanol / (coprostanol + 5β-stigmastanol) x 100% ^¶^	9% **✔** (7–13%)	14% **✔** (10–20%)	6% **✔** (5–8%)	7% **✔** (6–9%)	12% **✔** (11–13%)	16% **✔** (12–21%)	33% **✘** (29–38%)	69% **✘** (66–71)
VI	epi-5β-stigmastanol / 5β-stigmastanol + epicoprostanol / coprostanol	0.22 **✔** (0.18–0.29)	0.11 **✔** (0.07–0.16)	0.45 **✔** (0.36–0.55)	2.36 **✔** (2.08–2.71)	0.30 **✔** (0.27–0.32)	0.19 **✔** (0.12–0.30)	0.08 **✔** (0.04–0.13)	0.01 **✔** (0.01–0.01)

All ratios were calculated from the means of n = 3 laboratory replicates, except for the pigs (ratios were calculated from the means of the faecal steroid contents of Turopolje and Mangaliza pigs, n = 2 real replicates with each n = 3 laboratory replicates); range in parentheses

References for used ratios:

^†^ Bull et al., 1999;

^‡^ modified from Bull et al., 1999;

^§^ Grimalt et al., 1990;

^¶^ Leeming et al., 1997

Ratios for detection of faecal matter (No. I-IV):

**✔** = faecal input confirmed > 0.7;

**+/-** = faecal input can neither be confirmed nor excluded 0.3–0.7;

**✘** = faecal input should be excluded < 0.3

Ratios for source identification of faecal matter:

No. V: < 38% faeces of herbivores; > 73% human faeces

No. VI: > 1.2 horse faeces; < 0.8 no horse faeces;

**✔** source identification was possible

**✘** source identification was not possible.

A ratio from Bull et al. [47] using the typical human stanols, (coprostanol + epicoprostanol): (coprostanol + epicoprostanol + 5α-cholestanol), **ratio I**, and a modification of this ratio with the typical herbivore stanols (5β-stigmastanol + epi-5β-stigmastanol): (5β-stigmastanol + epi-5β-stigmastanol + 5α-stigmastanol), **ratio II**, performed best on our results (Table 4) and on those from the literature (S7 and S8 Tables), because it was possible to detect most of the livestock and human faeces as faecal material.

However, both ratios could not detect all of the analysed faeces as faecal material, regarding the usually applied threshold value of 0.7. This threshold value was originally established by Grimalt et al. [46] for a detection of human faecal matter in sediments by using ratios of 5β- to 5α-stanols and 5β- to 5α-stanones (Table 4, **ratios III** and **IV**) and was later on also applied to other ratios (**ratio I** and **II**). Yet, it has already failed in some studies, as it could not clearly indicate an input of human faecal matter (i.e. values >0.7) even in areas with a proven large faecal input [46,67]. For steroid contents of human faeces from our study and those from the literature **ratio III** worked well yielding values of >0.9, but for livestock faeces values ranged widely from 0.43 to 0.90 (Table 4) and from 0.41 to 0.89 (S7 and S8 Tables), respectively. In contrast, **ratio IV** failed completely for most livestock faeces and even for human faeces (Table 4, S7 and S8 Tables). Hence, for a detection of a faecal input potentially covering a wide range of possible faecal sources, **ratios I** and **II** are most suitable. Nevertheless, also enhanced contents of bile acids are a clear evidence, as bile acids are only produced by vertebrates [49–50; Table 3; S9 Table).

It is thus that for detection of human and livestock faeces in the environment, two aspects have to be taken into account. First, livestock faeces show smaller values of steroid ratios than human faeces (Table 4; S7 and S8 Tables). If they therefore fall short of the threshold value of 0.7 they will thus not be detected as faeces. This was e.g. the case for goat and geese faeces (ratio I and III). Second, for a detection of human and livestock faeces in archaeological soil material, a dilution as well as a degradation of the faecal markers have to be considered, as both aspects may lead to a failure of the applied ratios [2–4]. It is thus recommended that instead of using threshold values, the values of the applied ratios (or bile acid contents) should be compared with those from reference soils (control), thereby considering the local background contents of steroids and a possible degradation of the faecal markers [4,47]. In case of larger values of steroid ratios or bile acid contents compared to the control, a faecal input could thus be confirmed [2].

### Source identification of faecal matter

#### Differentiation between herbivore and omnivore faeces

Steroids have not only been used to detect a faecal input, but also to identify the faecal source [38,41–42,54,64,68]. However, so far it has only been possible to distinguish between faeces from pig, human and the group of herbivores by their steroid profile, but not to differentiate between faeces from different individual herbivore livestock species [27]. In order to set up a scheme for an identification of all common livestock and human faeces, we compared our results and those reported in literature. For this purpose we applied commonly used and new established stanol ratios for source identification of faecal residues, but complemented these assignments by additionally including bile acid contents and ratios. Here again we did not consider any ratios using Δ^5^-sterols due to their ubiquitous occurrence (see above; Fig 1).

Commonly used stanol ratios failed to identify all kind of faeces when using the proposed threshold values, for our data and those reported in the literature (Table 4; S7–S10 Tables). Only a ratio established 1997 by Leeming and co-authors [68], **ratio V**, i.e. coprostanol / (coprostanol + 5β-stigmastanol) x 100%, which relies on both 5β-stanols to distinguish between human and herbivore faeces (human >73%; herbivore <38%), performed well for our samples of herbivore faeces. Nevertheless, it falsely identified pig faeces as herbivore faeces, and failed to identify human faeces (Table 4) on the basis of the given threshold values. Similar observation could be made when applying **ratio V** on results reported in the literature yielding mean values of 34% (ranging from 17–54%), 47% (ranging from 43–51%), and 76% (ranging from 67–87%) for herbivore, pig and human faeces, respectively (S7 and S8 Tables), thereby also failing in the identification of some herbivore and human faecal samples.

**Fig 4:**
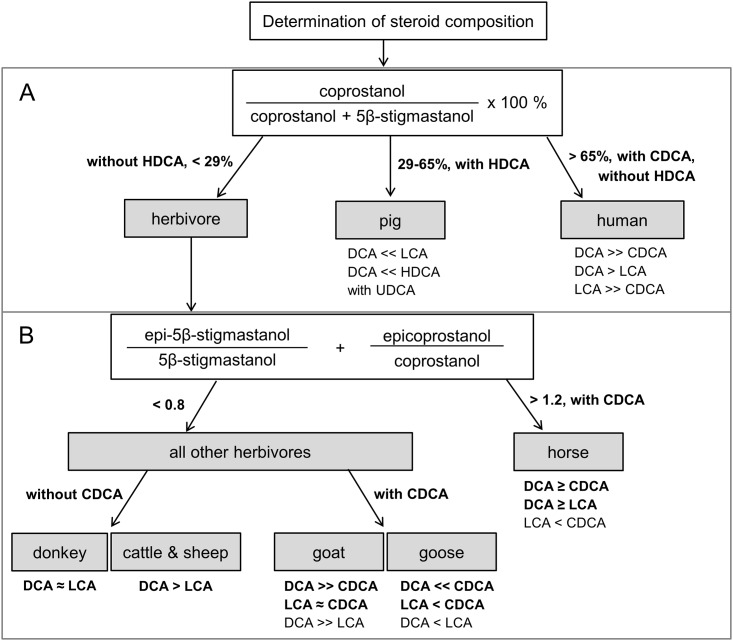
Criteria for the identification of pure livestock faeces by their steroid signature. Distinguishing parameters printed in bold type. A: Differentiation between herbivores, pig and human faeces, B: Differentiation between faeces of different herbivores. CDCA = chenodeoxycholic acid, DCA = deoxycholic acid, HDCA = hyodeoxycholic acid, LCA = lithocholic acid, UDCA = ursodeoxycholic acid.

Overall, for **ratio V**, our results corroborated with those of earlier publications showing increasing values in the order herbivore faeces (23% ± 15%) < pig faeces (42% ± 9%) < human faeces (74% ± 15%; Table 4; S7 and S8 Tables; [69]). However, the ranges overlapped between herbivore and pig faeces as well as between pig and human faeces; hence, further markers are needed for exact source assignment. For pig faeces the bile acid HDCA is a suitable marker, as it has not been found in any other animal faeces, yet (Table 3; [27,52,54]). We therefore propose a differentiation between herbivore, pig and human faeces by a modification of the threshold values of **ratio V** with values < 29% for herbivore, 29 ≤ ratio V ≤ 65% for pig, and values > 65% for human faeces, together with the presence of HDCA as an indication of pig faeces (Fig 4A).

#### Differentiation between faeces of herbivores

For a further differentiation between herbivore faeces, none of the ratios used to date turned out to be suitable (S10 Table; [38,44]). We thus established a new ratio (**ratio VI**), taking into account the—compared to all other human and livestock faeces—large epi-5β-stanols but equally large or smaller 5β-stanols contents of horse faeces (Table 2):

ratio VI=(epi-5β-stigmastanol/5β-stigmastanol)+(epicoprostanol/coprostanol)

With the help of this ratio it was possible to distinguish between faeces of horses and all other herbivores (and omnivores), as the ratio for horse faeces showed values between 2.1–2.7, without any overlap with values from other herbivore or omnivore faeces (0.01–0.6; Table 4). This finding was confirmed when applying the ratio to steroid contents of the study by Gill et al. [41], yielding values of 1.39 for horse faeces relative to values of 0.6–0.7 for cows and sheep (S7 Table). The only exception to this observation were larger **ratio VI** values for faeces from cows fed with silage (S2 and S7 Tables; [41–42]. We therefore excluded the samples from livestock animals that had not been fed with traditional fodder from our sample set and those from the literature from further considerations. This is in line with the archaeological context, because silage feeding only spread from the 1950´s on [70]. Hence, an input of faecal matter into soil from livestock fed with silage can be excluded for archaeological times and **ratio VI** remains valid for the archaeological context.

An increase in epi-5β-stanol relative to 5β-stanol contents has also been observed for faecal material exposed to anaerobic processes [35,42], but has also been discussed for the process of manure composting with compost piles showing aerobic as well as anaerobic micro-zones [15,71]. For a clear identification of faecal remains in archaeological soil material it is thus necessary to consider the archaeological finding and context and to validate the source assignment of **ratio VI** by additional markers, like bile acids. In doing so, and when combining our results with those published earlier [41,54], we suggest that horse faeces can be distinguished from those of other livestock when **ratio VI** exceeds a value of 1.2. Additionally, bile acid analyses can point to the presence of horse faeces, when deoxycholic (DCA) to chenodeoxycholic acid (CDCA) ratios and deoxycholic to lithocholic acid (LCA) ratios of DCA/CDCA = 0.8–2.1 and DCA/LCA = 1.0–3.4 can be observed, respectively (Fig 4B, Tables 3 and 4, S7–S9 Tables).

Also faeces from all other studied herbivores (comprising a **ratio VI** value <0.8; Table 4; Fig 4B) could subsequently be divided into further groups by the presence and ratios of different bile acids. Besides its occurrence in horse faeces, the presence of CDCA pointed to goose and goat faeces (Table 3; Fig 4B). The occurrence of CDCA in goose faeces is supported by a study of Hofmann et al. [72], who reported that CDCA was one important bilary bile acid of different wild geese species (subfamily *Anserinae*). Yet, we are not aware of any study analysing steroid contents of goat faeces. Hagey et al. [73] could also detect CDCA in the bile of cows and sheep, but usually only small amounts of bilary bile acids produced are excreted with the faeces [27,53]. Hence, it is plausible that neither we nor Tyagi et al. [54] detected CDCA in the faeces of cows. There is no comparable study, which analysed the faecal bile acids from sheep. Among the herbivores whose faeces contained CDCA (goats, geese, and horses), the contents of DCA and CDCA were nearly equally large in the horse faeces, in goat faeces the contents of DCA were about 30 times larger than those of CDCA, whereas geese faeces contained about 20 times smaller DCA than CDCA contents (Table 3; Fig 4B). Among the herbivores, whose faeces did not contain any CDCA, the faeces of the donkey showed nearly equally large amounts of DCA and LCA, which clearly distinguished them from faeces of cattle and sheep that showed both about five to ten times larger DCA to LCA contents (Table 3, S9 Table; Fig 4B).

It has to be noted that all faeces, those of herbivores and omnivores, comprised significant amounts of LCA and DCA (Table 3; S9 Table). The content ratio of DCA to LCA was—for our results and those in the literature—smallest for pig and geese faeces (0.01–0.4), intermediate for human, horse and donkey faeces (0.6–4.5), while cattle, sheep, and goat faeces exhibited the largest ratios (5–48; Table 3; S9 Table). The similarity of the ratios for humans and horses is in accordance to the literature ([54]; S9 Table), whereas donkey faeces have not been analysed on their bile acid contents, yet. In consequence, the earlier assumption that nearly equal contents of DCA and LCA can solely be attributed to human faecal matter [27] has to be revised, because the same is true for horse and donkey faeces.

In summary, faeces of different livestock could be distinguished by their 5β-stanol, epi-5β-stanol, and bile acid contents. Solely the faeces of sheep and cattle had very similar stanol and bile acid patterns and could therefore not be distinguished (Fig 4B). In any case, whenever a source identification of faeces is aimed for, it is necessary to combine the analyses of stanols and bile acids. Then, even faeces of goat and sheep could be distinguished. This is an important distinction, as the faeces of goats and sheep have a similar morphology [13], and as additionally their key skeletal elements are difficult to differentiate [74–76]. In the course of archaeological excavations it is therefore common to classify remains of goats and sheep as “ovicaprid” remains [77], as, up to now, a differentiation between both was hardly possible.

It has to be stressed that the above presented identification scheme (Fig 4) was established on the basis of pure faeces. For a mixed input of different faeces to soil, it must be considered that the values of the established ratios will likely shift.

### Application of established criteria for detection and identification of a faecal input into archaeological soil samples

After establishing criteria for detection and identification of a faecal input from pure livestock faeces (Table 4; Fig 4), we tested the applicability of these criteria for archaeological samples of different age but with known or supposed faecal input. First we analysed samples from a horse stable and a supposed cesspit from the Roman fort in **Dormagen**. We thus expected to find a chemical signal for horse faeces in the stable area and a signal for human faeces in the latrine. However, we also assumed that there might have been an input of pig faeces, as 16% of the animal bones that had been found in the course of an earlier excavation were pig bones, and as these livestock animals were—in contrast to cattle, sheep and goats—often kept inside a fort [78].

**Fig 5:**
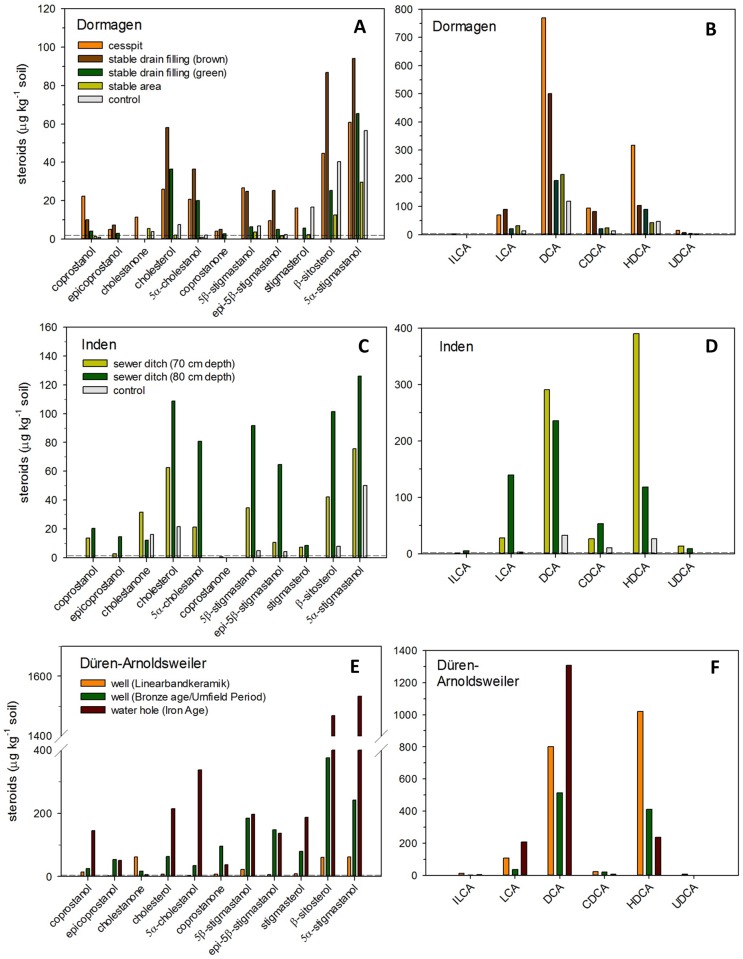
Δ^5^-sterol, stanol, stanone (A, C, E) and bile acid contents (B, D, F) of the archaeological soil samples from the sites Dormagen, Inden, and Düren-Arnoldsweiler (real replicates: n = 1). The dashed lines mark the limits of quantification. ILCA = isolithocholic acid, LCA = lithocholic acid, DCA = deoxycholic acid, CDCA = chenodeoxycholic acid, HDCA = hyodeoxycholic acid, UDCA = ursodeoxycholic acid; Legend of each site presented in Figs 5A, C, and E, respectively.

In all samples of the site Dormagen we detected coprostanol and 5β-stigmastanol. However, only in the samples “cesspit”, “stable drain with brown filling”, and “stable drain with green filling” the contents of coprostanol were above the routine limit of quantification (of 2 ng g^-1^ soil). The same was true for coprostanone, another significant steroid biomarker for faeces (Fig 5A). For the samples “cesspit” and “stable drain with brown filling”, ratio I [47] and ratio II (modified after Bull et al. [47]) showed distinctly larger values compared to the control sample, whereas for the sample “stable drain with green filling” only ratio I was enhanced (Table 5). All samples did contain, however, small amounts of bile acids. Total bile acid contents in the samples “cesspit” amounted to 1262 μg kg^-1^ soil, and in the “stable drain with brown filling” to 780 μg kg^-1^ soil. These amounts were two to four times larger than those of the samples “stable drain with green filling” and “stable area”, and four to seven times larger than those of the control (Fig 5B). But even for the samples “stable drain with green filling” as well as “stable area”, total bile acid contents were about two times larger than those of the control (Fig 5B).

**Table 5:** Steroid ratios for detection and identification of a faecal input applied on archaeological soil samples.

No.	Ratio	Dormagen					Inden			Düren-Arnoldsweiler		
		Cess-pit	Stable			Control	Sewer		Control	Well		
			Drain (brown filling)	Drain (green filling)	Stable area		70 cm depth	80 cm depth		LBK	Bronze Age	Iron Age
	**Ratios for mere detection of faecal matter**											
I	(coprostanol + epicoprostanol) / (5α-cholestanol + coprostanol + epicoprostanol) ^†^	0.57	0.32	0.26	0	0	0.43	0.30	0	0.89	0.70	0.37
II	(5β-stigmastanol + epi-5β-stigmastanol) / (5α-stigmastanol + 5β-stigmastanol + epi-5β-stigmastanol) ^‡^	0.37	0.35	0.15	0.11	0.14	0.37	0.55	0.15	0.31	0.58	0.18
	**Ratios for identification of faecal matter**											
V	coprostanol / (coprostanol + 5β-stigmastanol) x 100% ^§^	46%	29%	39%	0%	0%	28%	18%	0%	40%	12%	42%
VI	epi-5β-stigmastanol / 5β-stigmastanol + epicoprostanol / coprostanol	0.58	1.74	1.50	-	-	0.50	1.42	-	0.41	2.90	1.04
	**Bile acid ratios**											
	DCA / LCA	11	5.6	9.3	6.7	8.9	10	1.7	13	7.4	14.1	6.3
	DCA / CDCA	8.2	6.1	9.2	9.1	9.4	11	4.5	3.2	37	24	204
	CDCA / LCA	1.3	0.9	1.0	0.7	1.0	0.9	0.4	4.1	0.2	0.6	0.03
	HDCA / LCA	4.6	1.2	4.3	1.3	3.5	14	0.8	11	9.5	11	1.1

All ratios calculated from the means of n = 3 laboratory replicates; when steroid contents were below the routine quantification limit, they were treated as zero for the calculation of the ratios.

References for the used ratios:

^†^ Bull et al., 1999;

^‡^ modified from Bull et al., 1999;

^§^ Leeming et al., 1997

CDCA = chenodeoxycholic acid, DCA = deoxycholic acid, HDCA = hyodeoxycholic acid, LCA = lithocholic acid

According to the above defined criteria for detection of faecal matter in the environment (i.e. enhanced stanol ratios and / or enhanced bile acid contents compared to the control samples), a faecal input could be confirmed for the samples “cesspit” and “stable drain with brown filling”: both ratios for the detection of a faecal input as well as total bile acid contents were larger than in the control sample (Table 5). However, also for the sample “stable drain with green filling”, ratio I and total bile acid contents exceeded the values of the control, indicating a former input of faeces. For the sample “stable area”, only elevated bile acid contents (relative to the control) hinted at former faeces inputs (Table 5, Fig 4B). Hence, particularly bile acid contents, and, with one exception, also the stanol ratios provided geo-archaeological evidence of former inputs of faeces into this site.

In accordance with our assumption that not only horses but also pigs had been kept in the fort, we also tried to identify other faecal origins in the presumed input of a faecal mixture. Ratio V [68] yielded for both stable drain fillings values between ≥ 29% and ≤ 65%, indicating a former input of pig faeces (Table 5; Fig 4B). This assumption was confirmed by enhanced contents of hyodeoxycholic acid (HDCA) relative to the control (Figs 4B and 5). However, as pig faeces only contain very small amounts of deoxycholic acid (DCA) compared to HDCA (Table 3; S9 Table; Fig 5B), the large contents of DCA in both stable drain fillings compared to the contents of HDCA, pointed to an additional input of faeces from herbivores (Table 3; Fig 4B).

The application of ratio VI to both samples from the stable drain, for a further differentiation of the herbivore faeces, yielded values larger than 1.2 (Table 5). Together with the presence of chenodeoxycholic acid (CDCA), these enhanced values indicated indeed the former input of horse faeces (Fig 4B). However, as DCA/CDCA and DCA/LCA ratios were larger than those of pure horse and pig faeces (Tables 3 and 5), also a minor contribution of other herbivore faeces (cattle, sheep, goat, or donkeys) to the faecal input cannot be excluded.

For the sample “cesspit”, ratio V showed its maximum relative to all other samples from the site Dormagen. Noteworthy, the value of 46% for ratio V did not only point not to a pure input of human faeces but also to the additional input of herbivore or pig faecal matter (Table 5). The latter conclusion is supported by peaking amounts of HDCA, whereas the even larger amounts of DCA indicate additional input of either herbivore or human faeces (Fig 5B; Table 3). Due to the fact that in the sample “cesspit” i) largest contents of coprostanol and DCA could be found (also occurring with largest contents in human faeces; Fig 5A and 5B, Tables 2 and 3), ii) CDCA could be detected in largest contents (Fig 5A and 5B), and iii) ratio V yielded largest values (Table 4), is seems very likely that the additional faecal input consisted of human faeces. In this regard geochemistry is in line with former assumptions from geoarchaeology, supporting the input of human faeces to the “cesspit”, though with an additional—unexpected and large—input of pig faeces.

In summary, for the site Dormagen the detection of a faecal input in the cesspit and in both stable drain fillings was possible. Additionally, the supposed faecal input of human faeces for the sample “cesspit” and of horse faeces, for the stable drain fillings, could be substantiated. However, for both sample types (cesspit and stable drain) an additional input of pig faeces could be detected resulting in a mixed signal and leading to a shift in the results of all applied ratios. It seems likely that pigs were free roaming in the fort and it is hence plausible that molecular markers of their faeces could be detected in all samples [78].

For the site **Inden** two samples (in 70 cm and 80 cm depth) of a sewer ditch from a Roman “Villa Rustica” (dating to 0–450 AD) plus a control sample from outside the ditch were analysed. Regarding the historical context, different faecal inputs could be expected. In the first Roman occupation phase of the study site, horses were playing a major role in the livestock inventory, as this phase was characterized by military operations with cavalry. It is thus assumed that their breeding in the study region started from the Early Roman Age on (c. 0–70 AD; [78]). In the second occupation phase (especially during the Middle Imperial period, c. 70–260 AD), after military hostility ceased in the area and in the course of an intensification of agriculture, a shift in livestock inventory occurred, leading to a dominance of cattle for ploughing, draught and packing purposes, as well as for the great demand for leather and meat. The great number of cattle was followed by pigs (for meat production), sheep, and goats, with an increasing number of goats, as they were highly valued for their leather ([78–81]; for further information see also S3 Text). Hence, an input of horse faeces into the lower layer and an input of pig, cattle, sheep, and goat faeces for the upper layer of the sewer ditch could be expected. It was, however, not clear if also human faeces were part of the sewage flowing into the ditch.

Analyses of steroids revealed for both samples from inside the ditch larger contents of the faecal stanols (coprostanol and 5β-stigmastanol), 12 to 16 times larger total bile acid contents, and enhanced ratios for a detection of faecal matter (ratio I and II) compared with the control (Fig 5C and 5D, Table 5). There was thus a clear geochemical indication of an input of faecal matter into both layers of the ditch, which nicely confirmed the archaeological finding of a sewer ditch.

The sample from the upper (younger) layer showed that coprostanol accounted for 28% to the sum of coprostanol and 5ß-stigmastanol, whereas this contribution was only 18% for the sample of the lower (older) layer (ratio V, Table 5), both values still suggesting a faecal input of herbivores. However, larger contents of HDCA compared to the control also pointed to an input of pig faeces (Fig 4A), with the upper layer comprising about three times larger HDCA contents than the lower layer (Fig 5D). In line with the archaeological assumptions, both layers consisted thus indeed of a mixed sewage of pig and herbivore faeces, with a larger amount of pig faeces in the upper layer (also supported by a larger ratio V value for the upper layer). A major contribution of human faeces, however, is very unlikely as ratio V values were distinctly smaller than the threshold of 65% (Table 5; Fig 4A). Concerning the portion of herbivore faeces in this faecal mixture, larger CDCA contents in both layers than in the control pointed to an input of geese, goat and / or horse faeces (Figs 4B and 5D). For the lower layer the assumption of horse faeces was supported by ratio VI exceeding a value >1.2 (Table 5, Fig 4B) and by a DCA/LCA ratio of 1.7, being typical for horse faeces (Table 3; S9 Table). For the upper layer the large DCA/CDCA value was comparable to that of goat faeces, but an additional input of sheep, cattle and geese faeces could not be excluded, as well, as CDCA/LCA and DCA/LCA values should be smaller for a mixture of goat and pig faeces (Tables 3 and 5). With regard to the archaeological context, it is likely that mixtures of different herbivore faeces prevailed, as, e.g., sheep and goats were usually kept together in a flock [78].

In summary, steroid analysis could confirm the input of faeces to the site “Inden” and therefore the archaeological finding of a sewer ditch. Additionally it could confirm the archaeological assumption of an input of horse faeces for the lower (older) layer, and indicate an input of goat faeces for the upper (younger) layer, whereas there were only hints to an input of other herbivore faeces for the lower layer (i.e. cattle, sheep, and geese). For both layers steroid analysis could also reveal an input of pig faeces, with the younger layer comprising a larger input than the older one, which is in accordance to assumptions about the agricultural production during the Roman Age in the study site region (see also S3 Text). Indications for human faeces were not found, which reveals that steroid analyses may indeed be a useful tool not only for validating but also for specifying archaeological hypothesis at a given site.

For the site **Düren-Arnoldsweiler** three features from different epochs were excavated, one on-site well dating to the Linearbandkeramik (LBK), one off-site well dating to the Bronze Age / Urnfield Period and one water hole near to an Iron Age settlement (on-site). Concerning the historical and archaeological context of each well, different faecal inputs could be expected:

- For the **LBK well**, situated in a settlement, an input of human, pig, sheep, goat and cattle faeces could have occurred (but not of horse and goose faeces). Particularly human faeces should always be present in a settlement, while the detection of animal faeces would reflect the livestock inventory for the LBK time of the beginning of agriculture in Central Europe ([80,82]; for further information see also S3 Text).
- For the **off-site well, dating to the Bronze Age/Urnfield Period**, pollen analysis revealed its location on a pasture [59]. Concerning this location and the period of its construction and use, a faecal input of pigs, cattle, horses, sheep and goats seemed likely (but not of goose faeces). In addition to the livestock inventory present from the Neolithic, horses were also present, as their domestication had started from the 4th millennium BC on ([82]; S3 Text).
- For the **on-site water hole (Iron Age)** the increased livestock inventory made an input of cattle, pig, sheep, goat, horse, chicken and goose faecal matter possible. Here again also an input of human faecal matter seemed likely, due to the proximity to a settlement (S3 Text).

However, despite the above described historical and archaeological context, there were no further indications (like those for the samples from the sites Dormagen and Inden) for an input of faecal matter to the water hole and the wells. Due to a lacking of indications for a faecal input and, as well, of a control sample, we related steroid contents from this site to those from the sites Dormagen and Inden.

Analyses of steroids revealed increasing total contents of Δ^5^-sterols, stanols and stanones with decreasing age of the features. The c. 7000 year old on-site LBK well exhibited with 245 μg total Δ^5^-sterols, stanols and stanones per kg soil the smallest contents, the c. 3000 year old tree trunk well (off-site) showed with 1219 μg kg^-1^ already five times larger contents, and the c. 2500 year old water hole from the Iron Age (on-site) revealed with 4275 μg kg^-1^ the largest contents of all three features (Fig 5E). All steroid spectra were dominated by plant sterols and stanols (β-sitosterol, stigmasterol and 5α-stigmastanol), comprising 53–57% (well from LBK and Bronze Age, respectively) to 75% (water hole dating to Iron Age) of total Δ^5^-sterols, stanols and stanones. Nevertheless, also significant amounts of faecal stanols and their reduction products (coprostanol, 5β-stigmastanol, coprostanone, epicoprostanol, epi-5β-stigmastanol) could be detected, amounting to 12–34% of total Δ^5^-sterols, stanols, and stanones. Intriguingly, the tree trunk well and the water hole—although being much older—contained 2 to 25 larger content sums of faecal stanols and of their reduction products than all other faeces-containing samples from the sites Dormagen and Inden. As a much larger faecal input for the well samples compared to the latrine, stable, and sewage ditch samples from Dormagen and Inden seemed unlikely, we suggest that one reason for the higher contents were better preservation conditions of the filling material due to a location of the well fillings below the permanent groundwater table (see Materials and Methods; [56–57]).

This assumption is supported by the Bronze Age and the Iron Age well fillings showing largest content sums of Δ^5^-sterols, stanols, and stanones of all analysed features (Fig 5E, S11 Table).

For bile acid analysis, both on-site features (well from LBK and water hole from Iron Age) exhibited with 1879 μg kg^-1^ and 1758 μg kg^-1^ the largest total bile acid contents of all investigated archaeological samples, though also for the tree trunk well large total bile acid contents could be observed (985 μg kg^-1^; Fig 5F). These values were similar to the total bile acid contents of the Roman cesspit (1263 μg kg^-1^) and even larger than those of the Roman stable drain (326–780 μg kg^-1^) in Dormagen and the Roman sewage ditch (560–749 μg kg^-1^) of the site Inden (Fig 5B and 5D). Altogether, large contents of faecal stanols (and their reduction products) and bile acids, together with large ratios I and II, thus confirmed an input of faeces into all wells, as all values were larger or in a similar range compared to those from the other features from the sites Dormagen and Inden that contained faeces (Table 5; Fig 5). In contrast to sterol, stanol, and stanone contents, bile acid contents of all features from the site Düren-Arnoldsweiler were noticeably large, pointing to their larger resistance towards degradation in soil [2,51].

In accordance with our assumption that humans and a multitude of livestock could have contributed to the faecal input, the further identification of the faecal sources had to address mixed signals. Ratio V yielded 40–42% for the on-site features and 12% for the off-site well (Table 5), hinting at an input pig faeces or a mixed input of pig, human and herbivore faeces into the features located in close proximity to settlements and at an input of herbivore faeces into the well located on a pasture (Fig 4A). The presence of pig faeces was supported for all features by elevated contents of HDCA (Figs 4A and 5F).

For the oldest well (on-site well dating to LBK) we assumed—on basis of the archaeological context—a faecal input of pig, cattle, sheep, goats, and humans. It is therefore in accordance with our assumptions that ratio VI was too small to point to an input of horse faeces (Table 5; Fig 4B). As the occurrence of horse and goose faeces could be excluded (by ratio VI for horse and the historical context for both animals; see S3 Text), the detection of CDCA (a bile acid only being present in human, horse, goat and goose faeces; Table 3; Fig 4) supported an input of human and/or of goat faeces. Strong indications for human faeces were the elevated ratio V (similar to those of the cesspit sample from the site Dormagen, although lower than the threshold value of 65%) together with large amounts of DCA (Tables 3 and 5; Fig 5F), whereas an input of goat faeces was supported by a DCA/CDCA value smaller than that of humans (thereby pointing to an additional faecal CDCA source). Additionally, a larger DCA/LCA value compared to those of humans and pigs not only pointed to an input of goat faeces, but could also be attributed to an input of cattle and sheep faeces (Tables 3 and 5). An input of these herbivore faeces could have contributed to a ratio V value < 65%.

Overall, for the LBK well an input of pig faeces was confirmed, and there were strong indications for an input of human and goat faecal matter, with possible contributions also from cattle and/or sheep faeces. Hence, for this well-preserved sample biomarkers could provide information on faecal inputs despite its high age of above 7000 years.

For the off-site well, formerly located on a pasture and dating to the Bronze Age / Urnfield Period, the small ratio V pointed to an input of herbivore faeces, whereas the large ratio VI, together with the presence of CDCA, supported the hypothesis of an input of horse faeces (Table 5, Fig 4B). However, as DCA/LCA and DCA/CDCA ratios were too wide for the mere presence of horse faeces, other herbivores, like cattle, sheep or goats, likely contributed to the enhanced DCA contents (Tables 3 and 5). Altogether, and in accordance with the archaeological context, steroid analysis supported an input of pig and horse faeces into the off-site well. The data also pointed to an input of other faeces, which could have stemmed from cattle, sheep or goats based on the chemical signatures in the sample. Among the latter the assignment to cattle faeces is most likely, as cattle were the dominating livestock of the Bronze Age. It thus seems that cattle and horses had been penned on the—probably fenced—pasture at the same or different times; whereas the presence of domesticated pigs or wild boars at the watering place possibly occurred before or after the time the area had been fenced in (see also S3 Text).

Steroid analysis of the water hole filling (dating back to the Iron Age) showed the largest DCA contents and ratio V value (Table 5) of all features from the site Düren-Arnoldsweiler, together with the smallest HDCA contents. These findings pointed to an input of human faeces being even larger than that of pig faeces (Table 3; although ratio V was <65% due to a dilution of faecal material from pigs and herbivores, see below). Besides, as the DCA/LCA and DCA/CDCA values were larger than those for pure human faeces (Tables 3 and 5; S9 Table), additional inputs of DCA, e.g., from cattle faeces, likely occurred. This input of pig and herbivore faeces may have also contributed to a reduction of ratio V below 65%. Additional evidences for an input of horse faeces were not consistent: ratio VI value laid in-between the threshold values of 0.8 and 1.2 (Fig 4), and the smallest CDCA contents of all archaeological features (including the control samples) did not support indications for horse faeces (nor for goose faeces). Considering that the Δ^5^-sterol and 5α-stanol contents of this sample exceeded the values of all other analysed archaeological features, we assume that the water hole served as a waste pit during the time of its refilling. It was probably filled with plant (large β-sitosterol and stigmasterol contents) and animal debris (large cholesterol content) together with human, pig and cattle faeces. Transformation processes (preferentially occurring under anaerobic conditions) may then have reduced the Δ^5^-sterols to 5α-stanols, 5β-stanols, and epi-5β-stanols [35,42], i.e., the increased value of ratio VI may be explained by transformation processes rather than by the additional input of horse faeces.

All in all, bile acid analyses confirmed for all features from the site Düren Arnoldsweiler an input of pig faecal matter. For both on-site features stanol and bile acid patterns gave indications for an additional input of human and goat (LBK well) or cattle faeces (Iron Age water hole), whereas stanol and bile acid patterns pointed for the off-site well from the Bronze Age /Urnfield Period to an additional input of horse and cattle faeces. In this regard, also for site Düren Arnoldsweiler steroid analyses was consistent with assumptions from archaeology.

## Conclusion

Steroid analyses of 5β-stanols, epi-5β-stanols, and bile acids on faecal samples from old livestock breed—that had been fed exclusively with traditional fodder—allowed to identify nearly all investigated livestock faeces on the basis of their steroid signature. Most prominent was the distinction between sheep and goat faeces, as the remains of both animals can, up to now, only hardly be distinguished in archaeology due to their similar bone and faecal morphology.

By using a combination of stanol and bile acid analysis together with the application of existing and here newly introduced biomarker ratios, it was also possible to distinguish between different faecal inputs for archaeological samples. All steroid data generally fitted into the archaeological context. Nevertheless, multiple sources of faeces (and other debris) present, partly complicated the exact source assignment. As a result, the archaeological context became additionally important for the interpretation of the results.

Noteworthy, sterol, stanol, stanone, and bile acid contents could be detected and assigned to faecal sources in the archaeological soil samples despite the fact that they had received the faecal input 1600 to 7000 years ago. Concerning the application of steroid ratios derived from fresh faecal material on archaeological soil material, ageing or degradation processes of steroid biomarkers in soils did not seem to be very compound-specific and a faecal source detection and identification was therefore not significantly restricted. Yet, best source assignments were achieved from bile acids, which appear to be better preserved than other steroid groups. The overall best preservation conditions for steroids were in soil material that was buried below the groundwater table, pointing to the importance of submerged samples for geo-archaeological site reconstructions.

## Supporting Information

S1 Fig Sampling at the site Dormagen (sampling points marked with circles). Cesspit (A), stable drain with brown filling inside and green filling at the outsides (B), stable area in close proximity to the stable drain (C); photos: Archaeological Heritage Management Rhineland.
(PDF)

S2 Fig Stable drain from horse stables at the site Dormagen; photo LVR-LandesMuseum Bonn/LVR-Amt für Bodendenkmalpflege im Rheinland, from Müller et al. (1979) 49 fig. 26. Permission for publication obtained from the LVR-LandesMuseum Bonn/LVR-Amt für Bodendenkmalpflege im Rheinland.
(PDF)

S3 Fig Plan of the Roman fort in Dormagen. H = stable area, I = soldier barrack. Plan by Th. Becker 2006, LVR-Amt für Bodendenkmalpflege im Rheinland/Martin Wurzel Archäologie und Umwelttechnik GmbH (U. Wölfert, I. Grohmann), from Grohmann (2009) 88 fig. 93. Permission for publication obtained from the LVR-LandesMuseum Bonn/LVR-Amt für Bodendenkmalpflege im Rheinland.
(PDF)

S4 Fig Roman sewer ditch before sampling at the site Inden (photo: Archaeological Heritage Management Rhineland) (PDF)

S5 Fig Site Düren-Arnoldsweiler: well with box-shaped wooden lining (Linearbandkeramik; A), treetrunk well (Bronze Age /Urnfield Period; B), Roman water hole with wickerwork revetment (C); photos LVR-Amt für Bodendenkmalpflege im Rheinland/Martin Wurzel Archäologie und Umwelt-technik GmbH, Stahnsdorf; from Gerlach et al., 2011. Permission for publication obtained from the LVR-LandesMuseum Bonn/LVR-Amt für Bodendenkmalpflege im Rheinland.
(PDF)

S6 Fig Chromatogram of the Δ^5^-sterol, stanol and stanone standard solution. (PDF)

S7 Fig Chromatogram of the bile acid standard solution. (PDF)

S8 Fig Mass spectrum and structural formula of cholesterol trimethylsilyl ether. (PDF)

S9 Fig Mass spectrum and structural formula of β-sitosterol trimethylsilyl ether. (PDF)

S10 Fig Mass spectrum and structural formula of stigmasterol trimethylsilyl ether. (PDF)

S11 Fig Mass spectrum and structural formula of 5α-cholestanol trimethylsilyl ether. (PDF)

S12 Fig Mass spectrum and structural formula of coprostanol trimethylsilyl ether. (PDF)

S13 Fig Mass spectrum and structural formula of epicoprostanol trimethylsilyl ether. (PDF)

S14 Fig Mass spectrum and structural formula of 5α-stigmastanol trimethylsilyl ether. (PDF)

S15 Fig Mass spectrum and structural formula of 5β-stigmastanol trimethylsilyl ether. (PDF)

S16 Fig Mass spectrum and structural formula of epi-5β-stigmastanol trimethylsilyl ether. (PDF)

S17 Fig Mass spectrum and structural formula of cholestanone. (PDF)

S18 Fig Mass spectrum and structural formula of coprostanone. (PDF)

S19 Fig Mass spectrum and structural formula of 4-cholesten-3-one. (PDF)

S20 Fig Mass spectrum and structural formula of 6-ketocholestanol trimethylsilyl ether. (PDF)

S21 Fig Mass spectrum and structural formula of methylated and silylated chenodeoxycholic acid. (PDF)

S22 Fig Mass spectrum and structural formula of methylated and silylated deoxycholic acid. (PDF)

S23 Fig Mass spectrum and structural formula of methylated and silylated hyodeoxycholic acid. (PDF)

S24 Fig Mass spectrum and structural formula of methylated and silylated isolithocholic acid. (PDF)

S25 Fig Mass spectrum and structural formula of methylated and silylated lithocholic acid. (PDF)

S26 Fig Mass spectrum and structural formula of methylated and silylated ursodeoxycholic acid. (PDF)

S1 Table Studies on steroid contents of animal or human faecal matter. Methods for quantification and analysed steroids.
(PDF)

S2 Table Studies with quantified faecal steroid contents. Studied animal species and their diet.
(PDF)

S3 Table Old livestock breeds: history of the breed, fodder, breeders, and number of samples used for this study. (PDF)

S4 Table Studied compounds, molecular structures, retention times, and selected characteristic ion fragments. (PDF)

S5 Table Standard series for calibration (faeces). (PDF)

S6 Table Standard series for calibration (soil). (PDF)

S7 Table Sterol, stanol, and stanone contents of herbivore faeces (own data and data from literature). (PDF)

S8 Table Sterol, stanol, and stanone contents of omnivore faeces (data from literature) (PDF)

S9 Table Bile acid contents of omnivore and herbivore faeces (own data and data from literature) (PDF)

S10 Table Further steroid ratios for source identification of a faecal input applied on steroid contents of faecal samples from this study. (PDF)

S11 Table Sterol, stanol, and stanone contents of archaeologcial soil samples. (PDF)

S12 Table Bile acid contents of archaeologcial soil samples. (PDF)

S1 Text Further Information on archaeological soil samples (site Dormagen). (PDF)

S2 Text Further information on archaeological soil samples (site Düren-Arnoldsweiler). (PDF)

S3 Text Archaeological and historical context for the complete study site region. (PDF)

S4 Text References (Supporting Information). (PDF)
