# Supplementary material for: Steroid Biomarkers Revisited – Improved Source Identification of Faecal Remains in Archaeological Soil Material
Source: PLoS One. 2017 Jan 6;12(1):e0164882. doi: 10.1371/journal.pone.0164882 (PMC5217961; doi:10.1371/journal.pone.0164882)
Supplement: S1 Fig — Cesspit (A), stable drain with brown filling inside and green filling at the outsides (B), stable area in close proximity to the stable drain (C); photos: Archaeological Heritage Management Rhineland. (PDF) [file pone.0164882.s001.pdf]

Supporting Information

"Steroid Biomarkers Revisited – Improved Source Identification of Faecal Remains in Archaeological Soil Material"

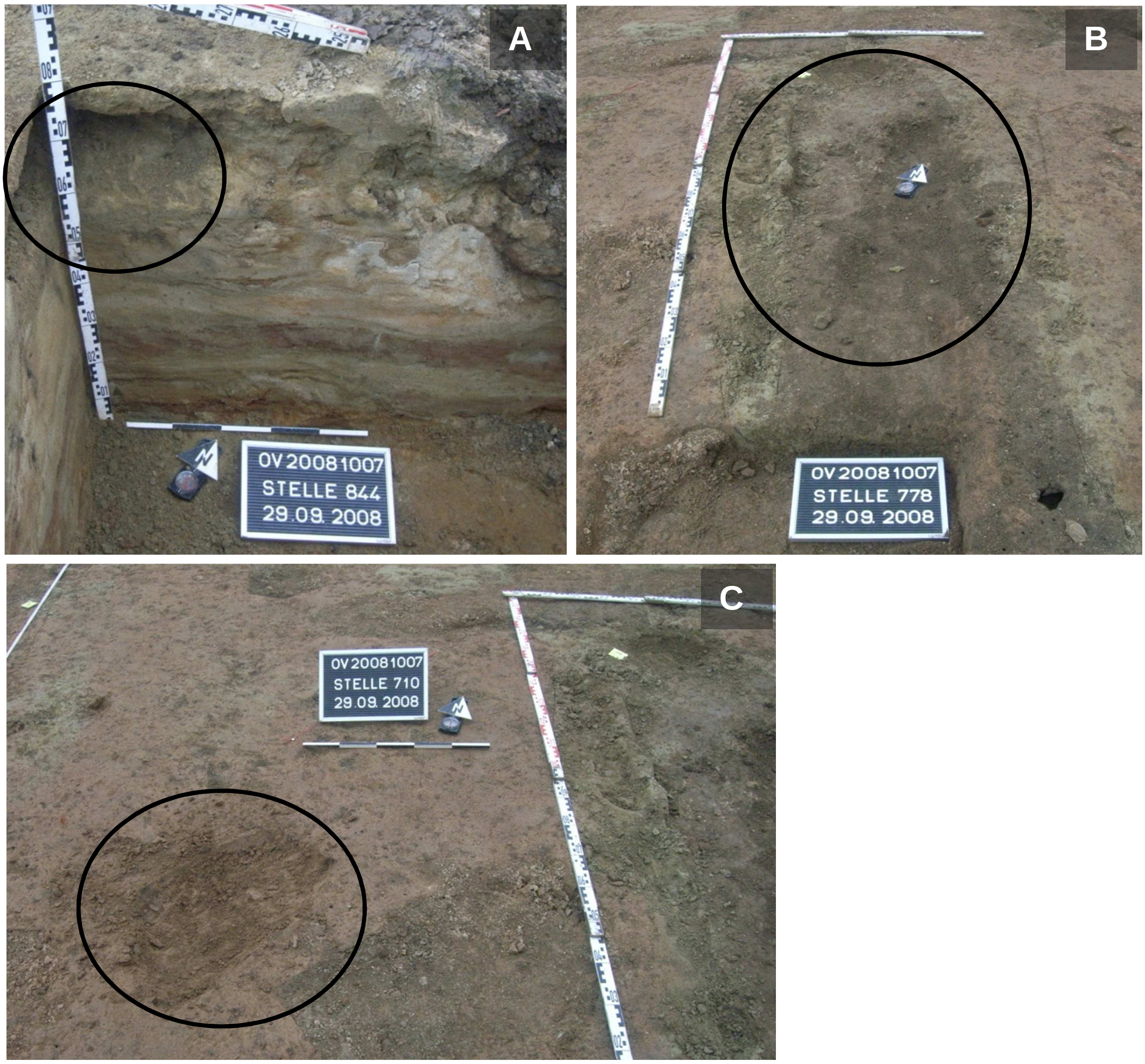

S1 Fig. Sampling at the site Dormagen (sampling points marked with circles). Cesspit (A), stable drain with brown filling inside and green filling at the outsides (B), stable area in close proximity to the stable drain (C); photos: Archaeological Heritage Management
