# Supplementary material for: Steroid Biomarkers Revisited – Improved Source Identification of Faecal Remains in Archaeological Soil Material
Source: PLoS One. 2017 Jan 6;12(1):e0164882. doi: 10.1371/journal.pone.0164882 (PMC5217961; doi:10.1371/journal.pone.0164882)
Supplement: S2 Fig — Permission for publication obtained from the LVR-LandesMuseum Bonn/LVR-Amt für Bodendenkmalpflege im Rheinland. (PDF) [file pone.0164882.s002.pdf]

Supporting Information

“Steroid Biomarkers Revisited – Improved Source Identification of Faecal Remains in Archaeological Soil Material”

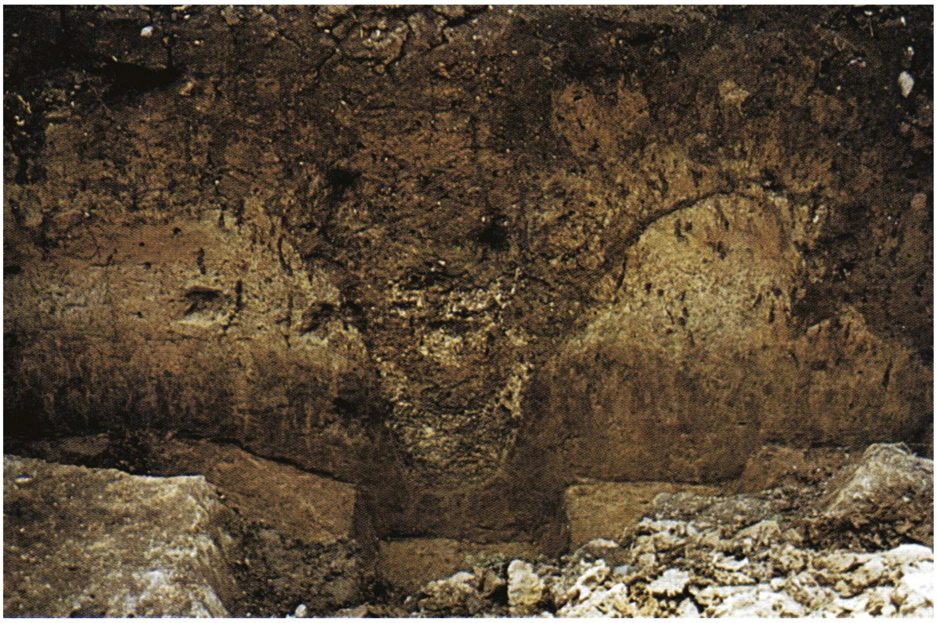

S2 Fig. Stable drain from horse stables at the site Dormagen; photo LVR-LandesMuseum Bonn/LVR-Amt für Bodendenkmalpflege im Rheinland, from Müller et al. (1979) 49 fig. 26.
Permission for publication obtained from the LVR-LandesMuseum Bonn/LVR-Amt für Bodendenkmalpflege im Rheinland.
