# Supplementary material for: Steroid Biomarkers Revisited – Improved Source Identification of Faecal Remains in Archaeological Soil Material
Source: PLoS One. 2017 Jan 6;12(1):e0164882. doi: 10.1371/journal.pone.0164882 (PMC5217961; doi:10.1371/journal.pone.0164882)
Supplement: S3 Fig — H = stable area, I = soldier barrack. Plan by Th. Becker 2006, LVR-Amt für Bodendenkmalpflege im Rheinland/Martin Wurzel Archäologie und Umwelttechnik GmbH (U. Wölfert, I. Grohmann), from Grohmann (2009) 88 fig. 93. Permission for publication obtained from the LVR-LandesMuseum Bonn/LVR-Amt für Bodendenkmalpflege im Rheinland. (PDF) [file pone.0164882.s003.pdf]

Supporting Information

"Steroid Biomarkers Revisited – Improved Source Identification of Faecal Remains in Archaeological Soil Material"

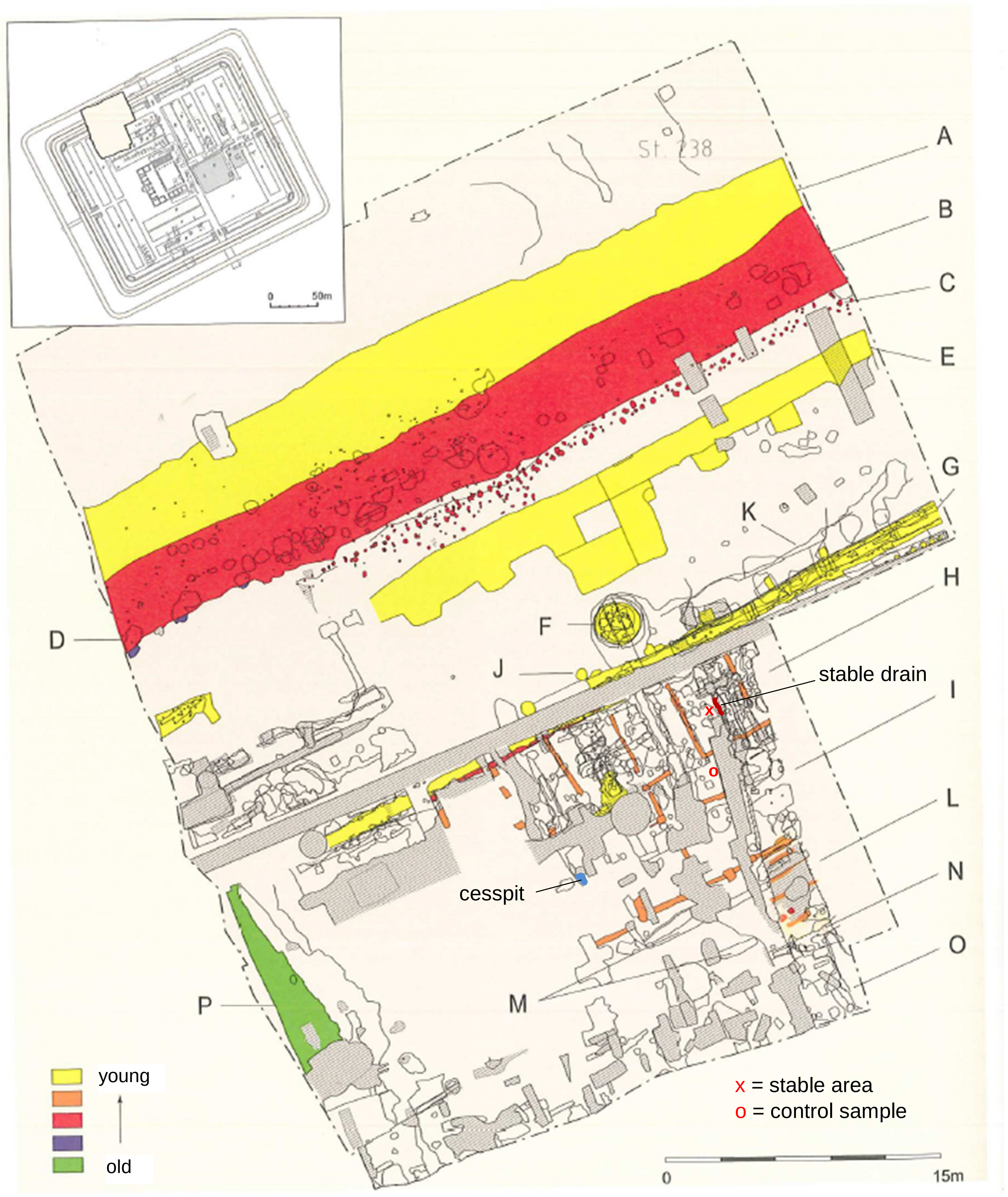

S3 Fig. Plan of the Roman fort in Dormagen. H = stable area, I = soldier barrack.
Plan by Th. Becker 2006, LVR-Amt für Bodendenkmalpflege im Rheinland/Martin Wurzel Archäologie und Umwelttechnik GmbH (U. Wölfert, I. Grohmann), from Grohmann (2009) 88 fig. 93. Permission for publication obtained from the LVR-LandesMuseum Bonn/LVR-Amt für Bodendenkmalpflege im Rheinland.
