# Supplementary material for: Steroid Biomarkers Revisited – Improved Source Identification of Faecal Remains in Archaeological Soil Material
Source: PLoS One. 2017 Jan 6;12(1):e0164882. doi: 10.1371/journal.pone.0164882 (PMC5217961; doi:10.1371/journal.pone.0164882)
Supplement: S5 Fig — Permission for publication obtained from the LVR-LandesMuseum Bonn/LVR-Amt für Bodendenkmalpflege im Rheinland. (PDF) [file pone.0164882.s005.pdf]

Supporting Information

“Steroid Biomarkers Revisited – Improved Source Identification of Faecal Remains in Archaeological Soil Material”

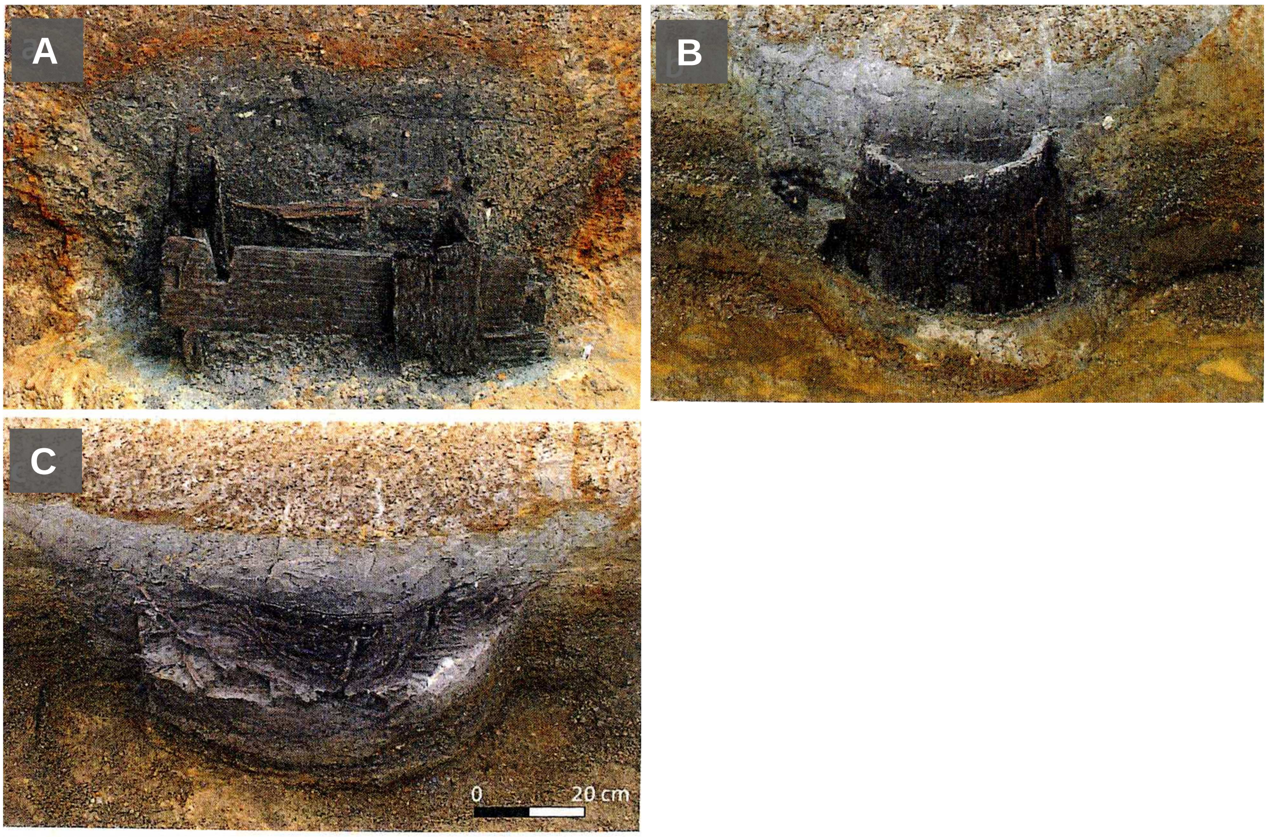

S5 Fig. Site Düren-Arnoldsweiler: well with box-shaped wooden lining (Linearbandkeramik; A), treetrunk well (Bronze Age /Urnfield Period; B), Roman water hole with wickerwork revetment (C); photos LVR-Amt für Bodendenkmalpflege im Rheinland/Martin Wurzel Archäologie und Umwelttechnik GmbH, Stahnsdorf; from Gerlach et al., 2011.
Permission for publication obtained from the LVR-LandesMuseum Bonn/LVR-Amt für Bodendenkmalpflege im Rheinland.
