# Supplementary material for: Steroid Biomarkers Revisited – Improved Source Identification of Faecal Remains in Archaeological Soil Material
Source: PLoS One. 2017 Jan 6;12(1):e0164882. doi: 10.1371/journal.pone.0164882 (PMC5217961; doi:10.1371/journal.pone.0164882)
Supplement: S6 Fig — (PDF) [file pone.0164882.s006.pdf]

Supporting Information

“Steroid Biomarkers Revisited – Improved Source Identification of Faecal Remains in Archaeological Soil Material”

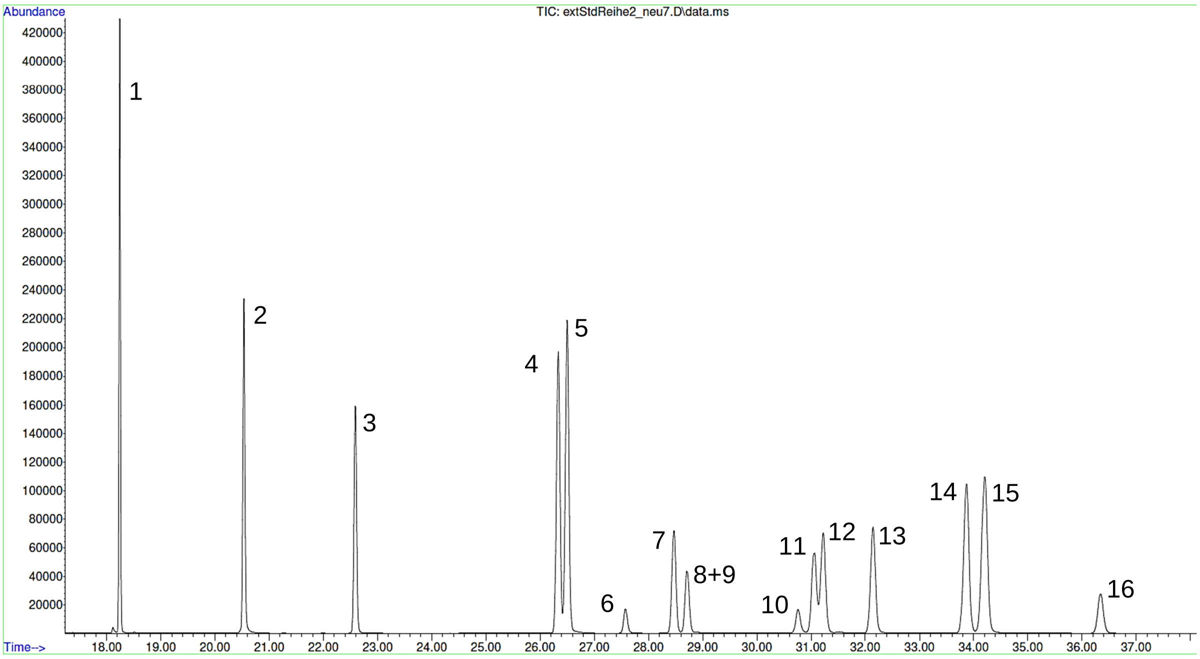

S6 Fig. Chromatogram of the Δ^5-sterol, stanol and stanone standard solution.

1 = Desoxypregnanolone (IS 1), 2 = Pregnanolone (IS 1), 3 = 5α-Cholestane (IS 2), 4 = Coprostanol, 5 = Epicoprostanol, 6 = Cholestanone, 7 = Cholesterol, 8 = 5α-Cholestanol, 9 = Coprostanone, 10 = 4-Cholesten-3-one, 11 = 5β-Stigmastanol, 12 = Epi-5β-Stigmastanol, 13 = Stigmasterol, 14 = β-Sitosterol, 15 = 5α-Stigmastanol, 16 = 6-Ketocholestanol
