# Supplementary material for: Steroid Biomarkers Revisited – Improved Source Identification of Faecal Remains in Archaeological Soil Material
Source: PLoS One. 2017 Jan 6;12(1):e0164882. doi: 10.1371/journal.pone.0164882 (PMC5217961; doi:10.1371/journal.pone.0164882)
Supplement: S7 Fig — (PDF) [file pone.0164882.s007.pdf]

Supporting Information

“Steroid Biomarkers Revisited – Improved Source Identification of Faecal Remains in Archaeological Soil Material”

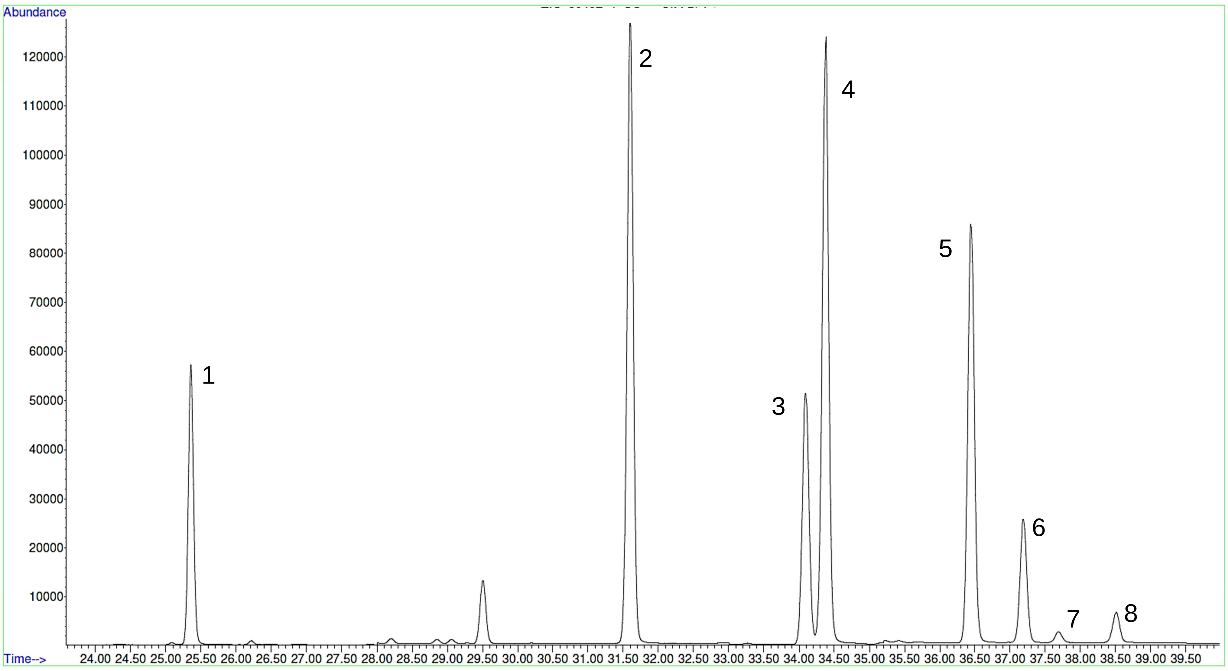

S7 Fig. Chromatogram of the bile acid standard solution.

1 = 5α-Cholestane (IS 2), 2 = Isodeoxycholic acid (IS 1), 3 = Isolithocholic acid, 4 = Lithocholic acid
5 = Deoxycholic acid, 6 = Chenodeoxycholic acid, 7 = Hyodeoxycholic acid, 8 = Ursodeoxycholic acid
