# Supplementary material for: Steroid Biomarkers Revisited – Improved Source Identification of Faecal Remains in Archaeological Soil Material
Source: PLoS One. 2017 Jan 6;12(1):e0164882. doi: 10.1371/journal.pone.0164882 (PMC5217961; doi:10.1371/journal.pone.0164882)
Supplement: S14 Fig — (PDF) [file pone.0164882.s014.pdf]

Supporting Information

"Steroid Biomarkers Revisited – Improved Source Identification of Faecal Remains in Archaeological Soil Material"

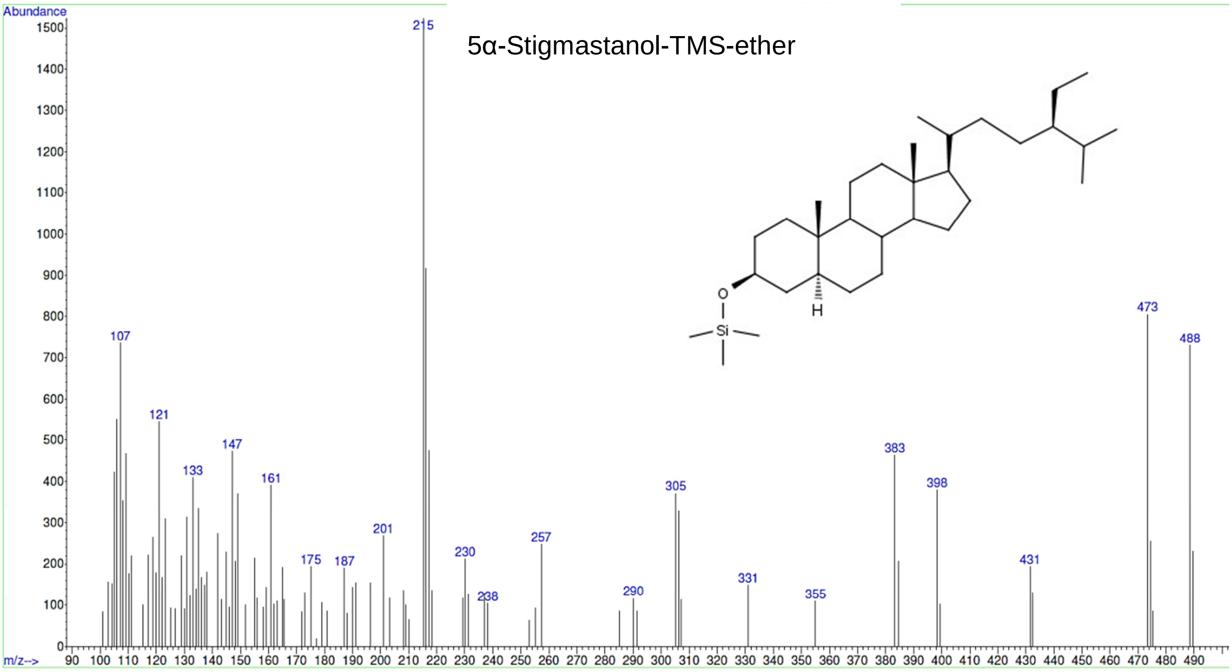

S14 Fig. Mass spectrum and structural formula of 5α-stigmastanol trimethylsilyl ether.
