# Supplementary material for: Steroid Biomarkers Revisited – Improved Source Identification of Faecal Remains in Archaeological Soil Material
Source: PLoS One. 2017 Jan 6;12(1):e0164882. doi: 10.1371/journal.pone.0164882 (PMC5217961; doi:10.1371/journal.pone.0164882)
Supplement: S23 Fig — (PDF) [file pone.0164882.s023.pdf]

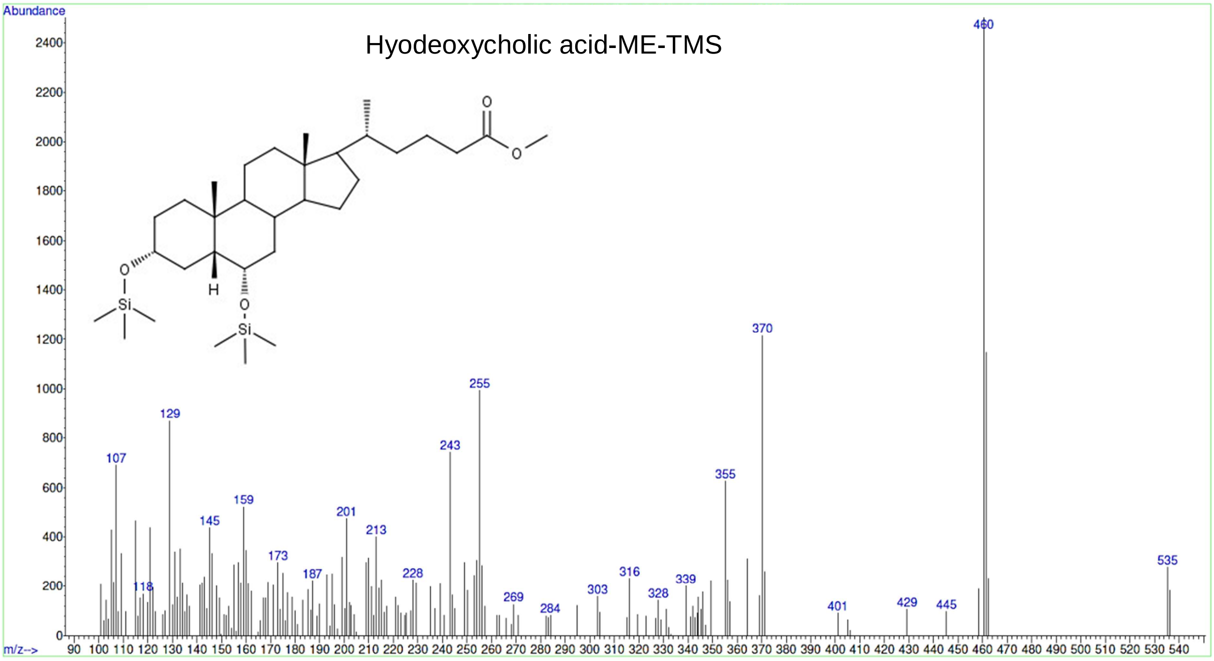

S23 Fig. Mass spectrum and structural formula of methylated and silylated hyodeoxycholic acid.
