# Supplementary material for: Steroid Biomarkers Revisited – Improved Source Identification of Faecal Remains in Archaeological Soil Material
Source: PLoS One. 2017 Jan 6;12(1):e0164882. doi: 10.1371/journal.pone.0164882 (PMC5217961; doi:10.1371/journal.pone.0164882)
Supplement: S1 Table — Methods for quantification and analysed steroids. (PDF) [file pone.0164882.s027.pdf]

Supporting Information

“Steroid Biomarkers Revisited – Improved Source Identification of Faecal Remains in Archaeological Soil Material”

S1 Table. Studies on steroid contents of animal or human faecal matter. Methods for quantification and analysed steroids.

Reference	GC analysis and quantification	Studied steroids					
		Δ^5-Sterols	5α-Stanols	5β-Stanols	Epi-5β-Stanols	Stanones	Bile acids
Batta et al., 2002	Calibration curves from standard solutions and internal standard; GC-FID						lithocholic acid<br>isodeoxycholic acid<br>deoxycholic acid<br>chenodeoxycholic acid<br>ursodeoxycholic acid
Derrien et al., 2011	Calibration curves from standard solutions (5β-stigmastanol, epi-5β-stigmastanol, and 5α-stigmastanol were quantified with the calibration curve of coprostanol); GC-MS: selected ion monitoring	cholesterol<br>stigmasterol<br>β-sitosterol	5α-cholestanol<br>5α-stigmastanol	coprostanol<br>5β-stigmastanol	epicoprostanol<br>epi-5β-stigmastanol		
Eneroth et al., 1964	Calibration curves from standard solutions	cholesterol<br>β-sitosterol		coprostanol<br>5β-stigmastanol		copro-stanone	
Gill et al., 2010	No information provided	cholesterol<br>stigmasterol<br>β-sitosterol	5α-cholestanol<br>5α-stigmastanol	coprostanol<br>5β-stigmastanol	epicoprostanol<br>epi-5β-stigmastanol		
Leeming et al., 1996	No information provided<br>(Scan)	cholesterol<br>stigmasterol<br>β-sitosterol	5α-cholestanol,<br>5α-stigmastanol	coprostanol<br>5β-stigmastanol	epicoprostanol<br>epi-5β-stigmastanol	copro-stanone	
Isobe et al., 2002	Calibration curves from standard solutions;<br>GC-MS measurement in selected ion monitoring	cholesterol<br>stigmasterol<br>β-sitosterol	5α-cholestanol<br>5α-stigmastanol	coprostanol	epicoprostanol	copro-stanone	
Reddy et al., 1998	Comparison of compound peak areas to peak area of internal standard of known concentration (androst-4-en-3,17-dione; methyl-7α,12α-dihydroxy-5β-cholan-3-oxo-24-oate)			coprostanol			lithocholic acid<br>deoxycholic acid<br>chenodeoxycholic acid
Shah et al. 2007	Internal standard and five-point calibration curves;<br>GC-MS measurement in selected ion monitoring	cholesterol<br>stigmasterol<br>β-sitosterol	5α-cholestanol	coprostanol<br>5β-stigmastanol	epicoprostanol		
Tyagi et al., 2007	Internal standard and eight-point calibration curves;<br>GC-MS measurement in selected ion monitoring	cholesterol<br>stigmasterol	5α-cholestanol<br>5α-stigmastanol	coprostanol	epicoprostanol		lithocholic acid<br>deoxycholic acid<br>chenodeoxycholic acid<br>hyodeoxycholic acid<br>ursodeoxycholic acid

Only compounds were considered that were also used in this study

Studies, in which steroid contents were not presented or not quantified: Evershed et al., 1997; Jardé et al., 2007; Bull et al., 1999; Standley et al., 2000
