# Supplementary material for: Steroid Biomarkers Revisited – Improved Source Identification of Faecal Remains in Archaeological Soil Material
Source: PLoS One. 2017 Jan 6;12(1):e0164882. doi: 10.1371/journal.pone.0164882 (PMC5217961; doi:10.1371/journal.pone.0164882)
Supplement: S2 Table — Studied animal species and their diet. (PDF) [file pone.0164882.s028.pdf]

Supporting Information

"Steroid Biomarkers Revisited – Improved Source Identification of Faecal Remains in Archaeological Soil Material"

S2 Table. Studies with quantified faecal steroid contents. Studied animal species and their diet.

Reference	Species*	Diet
Eneroth et al., 1964	humans (young men)	Omnivore, + corn oil/ + butter
Derrien et al., 2011	cows	grass + corn silage
	pigs	wheat + soybean (+ rapeseed)
Gill et al., 2010	cows	a) grass + hay<br>b) concentrate + silage<br>c) silage + concentrate
	sheep	grass
	horses	a) unknown<br>b) grass + hay + concentrates
Leeming et al., 1996	pigs	diet unknown
	humans	diet unknown
	cows	diet unknown
	horses	diet unknown
	sheep	diet unknown
Isobe et al., 2002	cows	diet unknown
Reddy et al., 1998	humans<br>(indian and caucasian woman)	vegetarian and omnivore
Shah et al., 2007	sheep (lambs)	diet unknown
	cows	diet unknown
	pigs	diet unknown
	donkeys	diet unknown
	horses	diet unknown
	humans	diet unknown
Tyagi et al., 2007	cow	diet unknown
	pig	diet unknown
	horse	diet unknown
	chicken	diet unknown

*comparable to those investigated in this study
