# Supplementary material for: Steroid Biomarkers Revisited – Improved Source Identification of Faecal Remains in Archaeological Soil Material
Source: PLoS One. 2017 Jan 6;12(1):e0164882. doi: 10.1371/journal.pone.0164882 (PMC5217961; doi:10.1371/journal.pone.0164882)
Supplement: S3 Table — (PDF) [file pone.0164882.s029.pdf]

Supporting Information

“Steroid Biomarkers Revisited – Improved Source Identification of Faecal Remains in Archaeological Soil Material”

S3 Table. Old livestock breeds: history of the breed, fodder, breeders, and number of samples used for this study.

Animal	History	Fodder	Number of Samples	Breeder	References
Rotes Höhenvieh (red cattle)	Breed of red cattle that has kept since several hundred years in the Central Uplands of Germany.	grass, leaves, silage	faeces from 5 different adult animals	Arche- und Naturlandhof Büning, Laer, Germany	Sambraus, 1999
Heck cattle	Cattle that was bred back to resemble the aurochs (*Bos primigenius*). For this breeding several domestic cattle breed were used (e.g., the Corsican Cattle, the Highland Cattle, the Hungarian Steppe Cattle).	grass, leaves	faeces from 5 different adult animals	Nature development area, Oostvaardersplassen, Lelystad, Netherlands	Sambraus, 2011
Konik horse	Old Polish horse landrace	grass, leaves	faeces from 5 different adult animals	Nature development area, Oostvaardersplassen, Lelystad, Netherlands	Sambraus, 2011
Poitou donkey	Originally bred in large parts of western France for the production of mules. According to written records the breed already existed in the 10th century.	grass	faeces from 1 adult animal (female)	Poultry and Mangalitza Pig Farm, Lautersheim, Germany	Sambraus, 2011,
Pomeranian Coarsewool Sheep	Old domestic sheep breed from the Pomerania and Silesian region (former Germany, now Poland). First bred in Silesia in the 13th century.	grass, hay, leaves, and fallen fruits	faeces from 5 different adult animals	Private breeder in Erftstadt, Germany	Sambraus, 1999, 2011
Black Forest Goat and White German Goat	Black forest goat<br>Bred since 1935 in Thuringia (Germany). Originates from local breeds and the Toggenburg goat.<br><br>White German Goat<br>Since the beginning of the 19th century there existed pure white goat breeds. In 1928 they were gathered under the name “White German Goat” and treated as a breed.	grass, herbs, and shrubs	faeces from 10 different adult animals	Private breeder in Vettweiß-Soller, Germany<br><br>Goats are kept for landscape management	Sambraus, 1999; 2011
German Laying goose	The breed originates from white feathered graylag geese. Since 1941 officially bred.	grass	faeces from 5 different adult animals	Private breeder in Vettweiß-Soller, Germany	GEH e.V., 2014
Turopolje Pig	Bacon pig that originally comes from Croatia. It was introduced there 1352. Turopolje are uncommonly hardy pigs, able to overwinter outdoors and forage extensively for themselves, being able to survive on a minimal diet.	fruits and vegetables	faeces from 5 different adult animals	Poultry and Mangalitza Pig Farm, Lautersheim, Germany	Arche Austria, 2014; Grunenfelder et al., 1994
Mangalitza Pig	Mangalitzas were developed from the older hardy types of Hungarian pig (Bakonyi and Szalontai breeds) crossed with the European Wild Boar and a Serbian breed (and later others like Alföldi) in Austro-Hungary (1833). 1927 Mangalitzas were officially recognised as a breed.	fruits and vegetables	faeces from 5 different adult animals	Poultry and Mangalitza Pig Farm, Lautersheim, Germany ernährt.	Oroian & Petrescu-Mag 2014
