# Supplementary material for: Steroid Biomarkers Revisited – Improved Source Identification of Faecal Remains in Archaeological Soil Material
Source: PLoS One. 2017 Jan 6;12(1):e0164882. doi: 10.1371/journal.pone.0164882 (PMC5217961; doi:10.1371/journal.pone.0164882)
Supplement: S4 Table — (PDF) [file pone.0164882.s030.pdf]

Supporting Information

“Steroid Biomarkers Revisited – Improved Source Identification of Faecal Remains in Archaeological Soil Material”

S4 Table. Studied compounds, molecular structures, retention times, and selected characteristic ion fragments.

Substance	Trivial name, molecular formula	Structure	Ret. time (min)	Characteristic ion fragments (m/z)
		**Δ^5-Sterols**		
Cholest-5-en-3β-ol	Cholesterol [b]<br>$C_{27}H_{46}O$	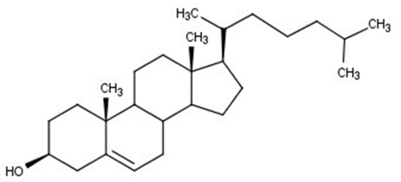	41.7	329, 368, 458
5-Stigmasten-3β-ol, 24-Ethyl-5-cholesten-3β-ol	β-Sitosterol [b]<br>$C_{29}H_{50}O$	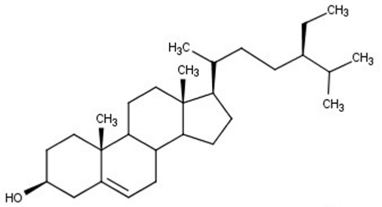	50.5	129, 396, 486
Stigmasta-5,22-dien-3β-ol	Stigmasterol [b]<br>$C_{29}H_{48}O$	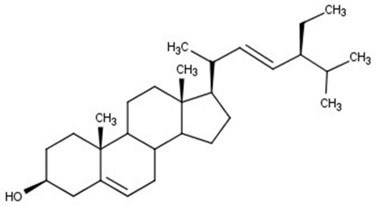	47.8	129, 394, 484
		**5α-Stanols**		
5α-Cholestan-3β-ol	5α-Cholestanol [b]<br>(Dihydrocholesterol)<br>$C_{27}H_{48}O$	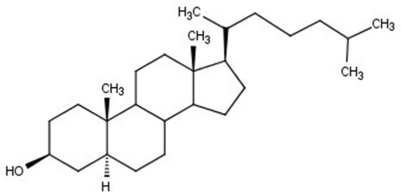	42.2	445
24α-Ethyl-5α-cholestan-3β-ol	5α-Stigmastanol [b]<br>(β-Sitostanol)<br>$C_{29}H_{52}O$	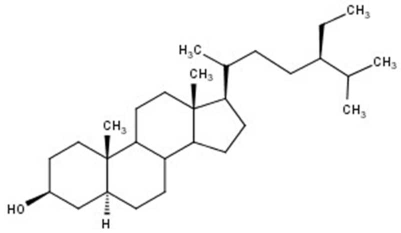	51.0	215, 383, 473
		**5β-Stanols**		
5β-Cholestan-3β-ol	Coprostanol [b]<br>$C_{27}H_{48}O$	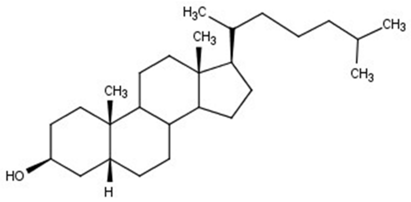	38.1	215, 257, 355, 370
24β-Ethyl-5β-cholestan-3β-ol	5β-stigmastanol [a,c]<br>(24-Ethylcoprostanol)<br>$C_{29}H_{52}O$	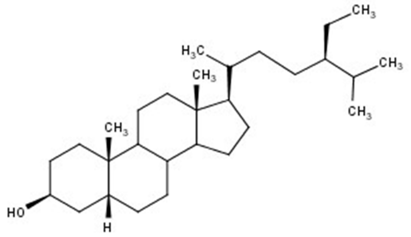	46.0	215, 257, 383, 398

[a] according to the designation of the National Institute of Standards and Technology (NIST)
[b] obtained from Sigma Aldrich (Taufkirchen, Germany)
[c] obtained from Chiron AS (Trondheim, Norway; supplier: Campro Scientific, Berlin, Germany)

S4 Table (continuation). Studied compounds, molecular structures, retention times, and selected characteristic ion fragments.

Substance	Trivial name, molecular formula	Structure	Ret. time (min)	Characteristic ion fragments (m/z)
		**Epi-5β-stanols**		
5β-Cholestan-3α-ol	Epicoprostanol [b] $C_{27}H_{48}O$	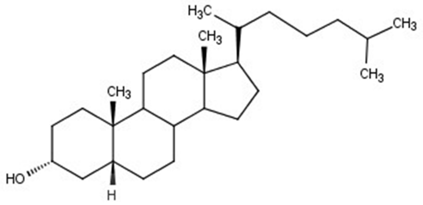	38.3	215, 257, 355, 370
24β-Ethyl-5β-cholestan-3α-ol	Epi-5β-stigmastanol [c] $C_{29}H_{52}O$	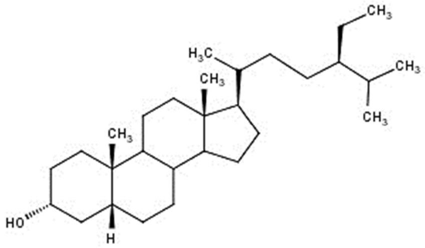	46.1	215, 257, 383, 398
		**Stanones**		
5α-Cholestan-3-one	Cholestanone [b] $C_{27}H_{46}O$	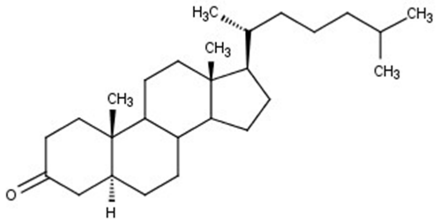	40.3	231, 316, 386
5β-Cholestan-3-one	Coprostanone [c] $C_{27}H_{46}O$	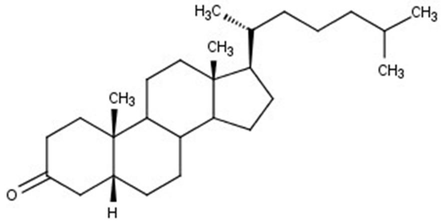	42.4	386
	4-Cholesten-3-one [b] $C_{27}H_{44}O$	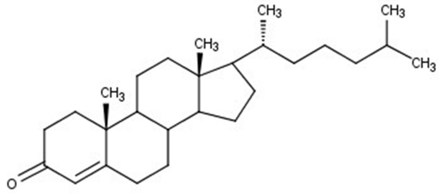	38.4	124, 229
3β-Hydroxy-5α-cholestan-6-one	6-Ketocholestanol [b] $C_{27}H_{46}O_2$	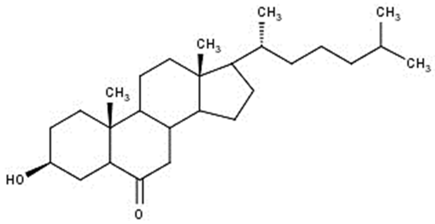	46.0	445, 459, 474

[b] obtained from Sigma Aldrich (Taufkirchen, Germany)
[c] obtained from Chiron AS (Trondheim, Norway; supplier: Campro Scientific, Berlin, Germany)

S4 Table (continuation). Studied compounds, molecular structures, retention times, and selected characteristic ion fragments.

Substance	Trivial name, molecular formula	Structure	Ret. time (min)	Characteristic ion fragments (m/z)
		**Bile acids**		
3β-Hydroxy-5β-cholanoic acid	Isolithocholic acid [d] (ILCA) $C_{24}H_{40}O_3$	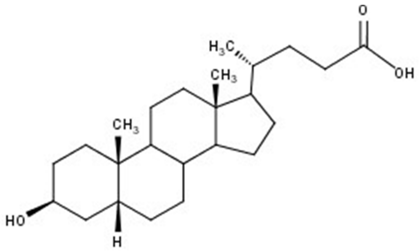	27.2	215, 257, 357
3α-Hydroxy-5β-cholanoic acid	Lithocholic acid [b] (LCA) $C_{24}H_{40}O_3$	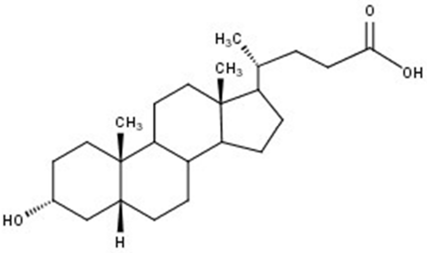	27.5	215, 257, 372
3α,12α-Dihydroxy-5β-cholanoic acid	Deoxycholic acid [b] (DCA) $C_{24}H_{40}O_4$	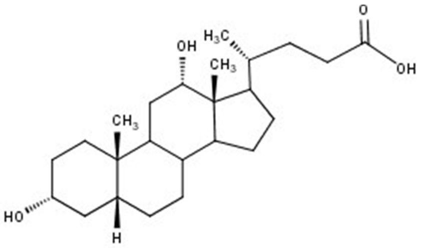	29.2	255, 345, 370
3α,7α-Dihydroxy-5β-cholanoic acid	Chenodeoxycholic acid [b] (CDCA) $C_{24}H_{40}O_4$	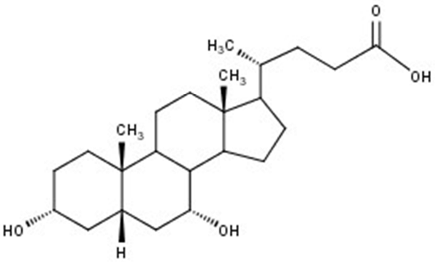	29.8	255, 355, 370
3α,6α-Dihydroxy-5β-cholanoic acid	Hyodeoxycholic acid [b] (HDCA) $C_{24}H_{40}O_4$	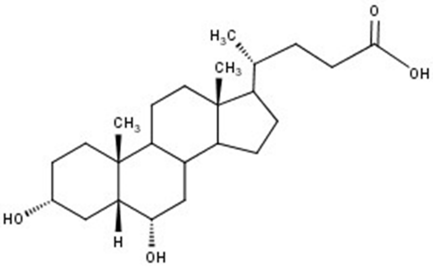	30.3	255, 355, 370
3α,7β-Dihydroxy-5β-cholanoic acid	Ursodeoxycholic acid [b] (UDCA) $C_{24}H_{40}O_4$	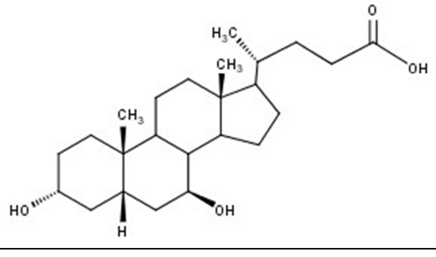	31.0	255, 370, 460

[b] obtained from Sigma Aldrich (Taufkirchen, Germany)
[d] obtained from Steraloids (Newport, RI, USA)

S4 Table (continuation). Studied compounds, molecular structures, retention times, and selected characteristic ion fragments.

Substance	Trivial name, molecular formula	Structure	Ret. time (min)	Characteristic ion fragments (m/z)
		**Internal standards**		
5β-Pregnan-3α-ol	Desoxypregnanolone [d,e]<br>$C_{21}H_{36}O$	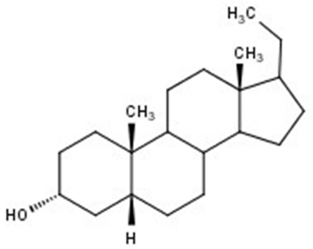	23.4	286, 361
5β-Pregnan-3α-ol-20-one	Pregnanolone [d,e]<br>$C_{21}H_{34}O_2$	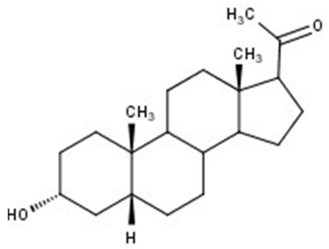	27.7	215, 300, 375
7α,12α-Dihydroxy-5β-cholanoic acid	Isodeoxycholic acid [d, e]<br>(IDCA)<br>$C_{24}H_{40}O_4$	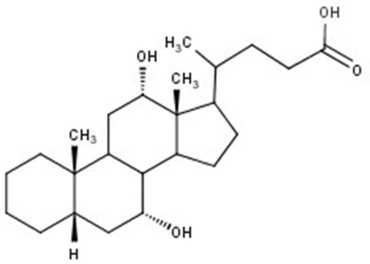	24.2	255, 355, 370
	5α-cholestane [d,f]<br>$C_{27}H_{48}$	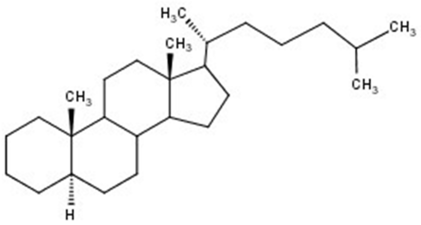	31.5[g] / 20.1[h]	217, 357, 372

[d] obtained from Steraloids (Newport, RI, USA)
[e] first internal standard (recovery standard)
[f] second internal standard
[g] measurement of sterols, stanols and stanones
[h] measurement of bile acids
