# Supplementary material for: Steroid Biomarkers Revisited – Improved Source Identification of Faecal Remains in Archaeological Soil Material
Source: PLoS One. 2017 Jan 6;12(1):e0164882. doi: 10.1371/journal.pone.0164882 (PMC5217961; doi:10.1371/journal.pone.0164882)
Supplement: S5 Table — (PDF) [file pone.0164882.s031.pdf]

Supporting Information

"Steroid Biomarkers Revisited – Improved Source Identification of Faecal Remains in Archaeological Soil Material"

S5 Table. Standard series for calibration (faeces)

**Substance**<br>Δ^5-sterols, stanols, and stanones	**Amounts spiked to standard series** (ng)	**Substance**<br>Bile acids	**Amounts spiked to standard series** (ng)
Cholesterol	0; 500; 1000; 2500; 5000	Isodeoxycholic acid, IS1	0; 50; 100;200; 500
β-Sitosterol	0; 1000; 2000; 5000; 10000	Isolithocholic acid	0; 25; 50; 100; 250
Stigmasterol	0; 500; 1000; 2500; 5000	Lithocholic acid	0; 50; 100;200; 500
Desoxypregnanolone, IS1	0; 1000; 2000; 5000; 10000	Deoxycholic acid	0; 100; 200; 400; 1000
Coprostanol	0; 500; 1000; 2500; 5000	Chenodeoxycholic acid	0; 50; 100;200; 500
5α-cholestanol	0; 500; 1000; 2500; 5000	Hyodeoxycholic acid	0; 50; 100;200; 500
Epicoprostanol	0; 500; 1000; 2500; 5000	Ursodeoxycholic acid	0; 50; 100;200; 500
5α-Stigmastanol	0; 1000; 2000; 5000; 10000		
5β-Stigmastanol	0; 1000; 2000; 5000; 10000		
Epi-5β-Stigmastanol	0; 500; 1000; 2500; 5000		
Pregnanolone, IS1	0; 1000; 2000; 5000; 10000		
5α-cholestan-3-one	0; 250; 500; 1250; 2500		
5β -cholestan-3-one	0; 250; 500; 1250; 2500		
6-Ketocholestanol	0; 250; 500; 1250; 2500		
4-Cholesten-3-one	0; 250; 500; 1250; 2500		
