# Supplementary material for: Steroid Biomarkers Revisited – Improved Source Identification of Faecal Remains in Archaeological Soil Material
Source: PLoS One. 2017 Jan 6;12(1):e0164882. doi: 10.1371/journal.pone.0164882 (PMC5217961; doi:10.1371/journal.pone.0164882)
Supplement: S7 Table — (PDF) [file pone.0164882.s033.pdf]

Supporting Information

"Steroid Biomarkers Revisited – Improved Source Identification of Faecal Remains in Archaeological Soil Material"

S7 Table. Sterol, stanol, and stanone contents of herbivore faeces (own data and data from literature).

Steroid (trivial name)	Sheep[1x] (n=1)	Sheep[2a] (n=1)	Sheep[3x] (n=6)	Horse[1x] (n=6)	Horse[2a] (n=1)	Horse[3x] (n=6)	Horse[4x] (n=26)	Donkey[3x] (n=6)
**Δ^5-Sterols (µg g^{-1})**								
Cholesterol	111 ±11‡	1754	327 ±88‡	39 ±9‡	52	16 ±2‡	1936 ±149‡	24 ±3‡
Stigmasterol	22 ±5	109	4 ±0.5	26 ±5	53	15 ±2	1340 ±1 00	15 ±2
β-Sitosterol	196 ±56	1506	22 ±3	218 ±48	505	59 ±8	nd	131 ±15
**5β-Stanols (µg g^{-1})**								
Coprostanol	170 ±27	1917	336 ±58	43 ±6	88	90 ±13	27 87 ±275	131 ±16
5β-Stigmastanol	246 ±69	2754	69 ±30	91 ±18	445	51 ±5	n d	83 ±10
**Epi-5β-stanols (µg g^{-1})**								
Epicoprostanol	16 ±3	268	10 ±7	5 ±1	62	22 ±3	2129 ±2 03	12 ±1
Epi-5β-stigmastanol	67 ±13	1467	nd	0 ±0	303	nd	nd	nd
**5α-Stanols (µg g^{-1})**								
5α-Cholestanol	49 ±6	888	42 ±12	25 ±5	56	32 ±2	1791 ± 111	20 ±2
5α-Stigmastanol	135 ±28	2304	nd	50 ±9	295	nd	4932 ±403	nd
**Stanones (µg g^{-1})**								
Coprostanone	17 ±2	nd	nd	7 ±2	nd	nd	nd	nd
Cholestanone	nd	nd	nd	nd	nd	nd	nd	nd
**Ratios**								
No. I	0.79 ✓	0.71 ✓	0.89 ✓	0.66 +/-	0.73 ✓	0.78 ✓	0.73 ✓	0.88 ✓
No. II	0.70 +/-	0.65 +/-	-	0.65 +/-	0.72 ✓	-	-	-
No. III	0.78 ✓	0.68 +/-	0.89 ✓	0.63 +/-	0.61 +/-	0.73 ✓	0.61 +/-	0.86 ✓
No. IV	-	-	-	-	-	-	-	-
No. V	41% ✗ (31-53%)	41% ✗	83% ✗ (74-91%)	32% ✓ (25-40%)	17% ✓	64% ✗ (58-69%)	-	61% ✗ (55-67%)
No. VI	0.37 ✓ (0.2-0.6)	0.67 ✓	-	0.12 ✗ (0.1-0.2)	1.39 ✓	-	-	-

nd = not determined; † mean ±standard deviation; ‡ mean ±standard error

We only considered studies, in which steroid contents were presented and quantified (not considered: Bull et al., 1999; Evershed et al., 1997; Jardé et al., 2007; Standley et al., 2000)

Cited studies: 1: Leeming et al., 1996; 2: Gill et al., 2010; 3: Shah et al., 2007 (µg g^{-1} wet weight basis); 4: Tyagi et al., 2007;

3: Results from Shah et al. (2007) presented here but excluded from further discussion due to extremely small 5β-stigmastanol contents compared to all other studies

Diet of the studied animals: x = no information provided; a = grass + hay (+concentrates);

Ratios for a detection of faecal matter (and reference):

No. I: (coprostanol + epicoprostanol) / (5α-cholestanol + coprostanol + epicoprostanol) (Bull et al., 1999);
No. II: (5β-stigmastanol + epi-5β-stigmastanol) / (5α-stigmastanol + 5β-stigmastanol + epi-5β-stigmastanol) (modified from Bull et al., 1999);
No. III: coprostanol / (5α-cholestanol + coprostanol) (Grimalt et al., 1990);
No. IV: coprostanone / (cholestanone + coprostanone) (Grimalt et al., 1990)

Threshold values (Ratios No. I-IV):
>0.7 faecal input confirmed ✓; 0.3-0.7 faecal input can neither be confirmed nor excluded +/-; <0.3 faecal input should be excluded ✗

Ratios for a source identification of faecal matter:

No. V: coprostanol / (coprostanol + 5β-stigmastanol) x 100% (Leeming et al., 1997)
No. VI: epi-5β-stigmastanol / 5β-stigmastanol + epicoprostanol / coprostanol

Threshold values (Ratios No. V-VIII):
No. V: < 38% faeces of herbivores; > 73% human faeces
No. VI: > 1.2 horse faeces; < 0.8 no horse faeces

✓ source identification was possible; ✗ source identification was not possible.

S7 Table (continuation). Sterol, stanol, and stanone contents of herbivore faeces (own data and data from literature)

**Steroid (trivial name)**	**Cow**[5b] (n=1)	**Cow**[1x] (n=6)	**Cow**[2a] (n=1)	**Cow**[2b] (n=2)	**Cow**[3x] (n=6)	**Cow**[4x] (n=24)	**Cow**[6x] (n=1)	**Cow**[7b] (n=6)
**Δ^5-Sterols ($\mu g\ g^{-1}$)**								**Sterols (%)**
Cholesterol	262 ±88[†]	201 ±53[‡]	155	281 ±66[†]	90 ±54[‡]	3774 ±247[‡]	223 ±8[†]	8.5
Stigmasterol	53 ±21	21 ±7	35	33 ±23	2 ±0.3	490 ±20	66 ±3	4.5
β-Sitosterol	263 ±82	187 ±34	360	360 ±7	11 ±0.4	nd	196 ±8	8.5
**5β-Stanols ($\mu g\ g^{-1}$)**								**5β-Stanols (%)**
Coprostanol	252 ±80	213 ±72	132	274 ±15	72 ±38	3658 ±264	203 ±9	6
5β-Stigmastanol	1940 ±628	184 ±41	821	635 ±32	6 ±1	nd	nd	9
**Epi-5β-stanols ($\mu g\ g^{-1}$)**								**Epi-5β-stanols (%)**
Epicoprostanol	97 ±14	15 ±6	15	111 ±1	0.7 ±0.4	543 ±54	54 ±2	5
Epi-5β-stigmastanol	1110 ±223	0 ±0	380	747 ±30	nd	nd	nd	20
**5α-Stanols ($\mu g\ g^{-1}$)**								**5α-Stanols (%)**
5α-Cholestanol	106 ±23	69 ±24	57	113 ±16	9 ±5	1791 ±111	128 ±5	8.5
5α-Stigmastanol	1052 ±150	210 ±23	504	212 ±157	nd	1133 ±65	377 ±19	15
**Stanones ($\mu g\ g^{-1}$)**								**Stanones (%)**
Coprostanone	54 ±17	19 ±7	nd	nd	nd	nd	84 ±3	nd
Cholestanone	68 ±25	nd	nd	nd	nd	nd	nd	nd
**Ratios**								
No. I	0.77 ✓	0.77 ✓	0.72 ✓	0.77 ✓	0.39 +/-	0.70 +/-	0.67 +/-	0.55 +/-
No. II	0.74 ✓	0.47 +/-	0.70 +/-	0.87 ✓	-	-	-	0.66 +/-
No. III	0.70 +/-	0.76 ✓	0.70 +/-	0.71 ✓	0.89 ✓	0.67 +/-	0.61 +/-	0.41 +/-
No. IV	0.44 +/-	-	-	-	-	-	-	-
No. V	11%<br>(6-20%)	54% ✗<br>(39-67%)	14%	30%<br>(28-32%)	92% ✗<br>(83-96%)	-	-	40% ✗
No. VI	0.96 ✗<br>(0.6-1.7)	0.07 ✓<br>(0.03-0.2)	0.58 ✓	1.58 ✗<br>(1.5-1.7)	-	-	-	3.1 ✗

nd = not determined; [†] mean ±standard deviation; [‡] mean ±standard error

We only considered studies, in which steroid contents were presented and quantified (not considered: Bull et al., 1999; Evershed et al., 1997; Jardé et al., 2007; Standley et al., 2000)

Cited studies: 1: Leeming et al., 1996; 2: Gill et al., 2010; 3: Shah et al., 2007 ($\mu g\ g^{-1}$ wet weight basis); 4: Tyagi et al., 2007; 5: Red cattle (own data, composite sample of n=5); 6: Isobe et al., 2002; 7: Derrien et al., 2011 (only fresh faeces considered)

3: Results from Shah et al. (2007) presented here but excluded from further discussion due to extremely small 5β-stigmastanol contents compared to all other studies

Diet of the studied animals: x = no information provided; a = grass + hay (+ concentrates); b = silage (+ concentrates or + grass)

Ratios for a detection of faecal matter (and reference):
No. I: (coprostanol + epicoprostanol) / (5α-cholestanol + coprostanol + epicoprostanol) (Bull et al., 1999);
No. II: (5β-stigmastanol + epi-5β-stigmastanol) / (5α-stigmastanol + 5β-stigmastanol + epi-5β-stigmastanol) (modified from Bull et al., 1999);
No. III: coprostanol / (5α-cholestanol + coprostanol) (Grimalt et al., 1990);
No. IV: coprostanone / (cholestanone + coprostanone) (Grimalt et al., 1990):

Threshold values (Ratios No. I-IV):
>0.7 faecal input confirmed ✓; 0.3-0.7 faecal input can neither be confirmed nor excluded +/-; <0.3 faecal input should be excluded ✗

Ratios for a source identification of faecal matter:
No. V: coprostanol / (coprostanol + 5β-stigmastanol) x 100% (Leeming et al., 1997)
No. VI: epi-5β-stigmastanol / 5β-stigmastanol + epicoprostanol / coprostanol

Threshold values (Ratios No. V-VIII):
No. V: < 38% faeces of herbivores; > 73% human faeces (Leeming et al., 1997)
No. VI: > 1.2 horse faeces; < 0.8 no horse faeces

✓ source identification was possible; ✗ source identification was not possible.
