# Supplementary material for: Steroid Biomarkers Revisited – Improved Source Identification of Faecal Remains in Archaeological Soil Material
Source: PLoS One. 2017 Jan 6;12(1):e0164882. doi: 10.1371/journal.pone.0164882 (PMC5217961; doi:10.1371/journal.pone.0164882)
Supplement: S8 Table — (PDF) [file pone.0164882.s034.pdf]

Supporting Information

“Steroid Biomarkers Revisited – Improved Source Identification of Faecal Remains in Archaeological Soil Material”

S8 Table. Sterol, stanol, and stanone contents of omnivore faeces (data from literature)

Steroid (trivial name)	Chicken[1x] (n=6)	Chicken[4x] (n=17)	Pig[1x] (n=6)	Pig[3x] (n=6)	Pig[4x] (n=26)	Pig[7c] (n=10)
		**Δ^5-Sterols (µg g^{-1})**				**Sterols (%)**
Cholesterol	219 ±41‡	5408 ±785‡	127 ±27‡	69 ±5‡	3606 ±357‡	11%
Stigmasterol	40 ±7	2872 ±380	0	7 ±1	1710 ±99	2%
β-Sitosterol	331 ±17	nd	139 ±10	151 ±11	nd	13.5%
		**5β-Stanols (µg g^{-1})**				**5β-Stanols (%)**
Coprostanol	12 ±7	1971 ±437	353 ±76	530 ±61	8802 ±4 79	15%
5β-Stigmastanol	58 ±5	nd	334 ±45	183 ±33	nd	20%
		**Epi-5β-stanols (µg g^{-1})**				**Epi-5β-stanols (%)**
Epicoprostanol	1 ±1	62 ±14	25 ±8	88 ±30	1427 ±151	2. 5%
Epi-5β-stigmastanol	0	nd	0 ±0	nd	nd	3%
		**5α-Stanols (µg g^{-1})**				**5α-Stanols (%)**
5α-Cholestanol	12 ±4	287 ±445	45 ±13	63 ±20	1668 ±199	8.5%
5α-Stigmastanol	92 ±10	4685 ±617	127 ±17	nd	14329 ±658	10%
		**Stanones (µg g^{-1})**				**Stanones (%)**
Coprostanone	3 ±2	nd	11 ±4	nd	nd	nd
Cholestanone	nd	nd	nd	nd	nd	nd
			**Ratios**			
No. I	0.52 **+/-**	0.88 ✓	0.89 ✓	0.91 ✓	0.86 ✓	0.67 **+/-**
No. II	0.39 **+/-**	-	0.72 ✓	-	-	0.70 **+/-**
No. III	0.50 **+/-**	0.87 ✓	0.89 ✓	0.89 ✓	0.84 ✓	0.64 **+/-**
No. IV	-	-	-	-	-	-
No. V	17% ✓ (7-26%)	-	51% (42-60%)	74% (68-80%)	-	43%
No. VI	0.08 ✓ (0-0.4)	-	0.07 ✓ (0.04-0.12)	-	-	0.32 ✓

nd = not determined; † mean ± standard deviation; ‡ mean ± standard error

We only considered studies, in which steroid contents were presented and quantified (not considered: Bull et al., 1999; Evershed et al., 1997; Jardé et al., 2007; Standley et al., 2000)

Cited studies: 1: Leeming et al., 1996; 3: Shah et al., 2007 (µg g^{-1} wet weight basis); 4: Tyagi et al., 2007; 7: Derrien et al., 2011 (only fresh faeces considered);

3: Results from Shah et al. (2007) presented here but excluded from further discussion due to extremely small 5β-stigmastanol contents compared to all other studies

Diet of the studied animals: x = no information provided; c = wheat, soybean, rapeseed;

Ratios for a detection of faecal matter (and reference):

No. I: (coprostanol + epicoprostanol) / (5α-cholestanol + coprostanol + epicoprostanol) (Bull et al., 1999);
No. II: (5β-stigmastanol + epi-5β-stigmastanol) / (5α-stigmastanol + 5β-stigmastanol + epi-5β-stigmastanol) (modified from Bull et al., 1999);
No. III: coprostanol / (5α-cholestanol + coprostanol) (Grimalt et al., 1990);
No. IV: coprostanone / (cholestanone + coprostanone) (Grimalt et al., 1990):

Threshold values (Ratios No. I-IV):
>0.7 faecal input confirmed ✓; 0.3-0.7 faecal input can neither be confirmed nor excluded **+/-**; <0.3 faecal input should be excluded ✖

Ratios for a source identification of faecal matter:

No. V: coprostanol / (coprostanol + 5β-stigmastanol) x 100% (Leeming et al., 1997)
No. VI: epi-5β-stigmastanol / 5β-stigmastanol + epicoprostanol / coprostanol

Threshold values (Ratios No. V-VIII):
No. V: < 38% faeces of herbivores; > 73% human faeces (Leeming et al., 1997)
No. VI: > 1.2 horse faeces; < 0.8 no horse faeces
✓ source identification was possible; ✖ source identification was not possible.

S8 Table (continuation). Sterol, stanol, and stanone contents of omnivore faeces (data from literature)

Steroid (trivial name)	Human[1x] (n=6)	Human[3x] (n=7)	Human[8d] (n=18)	Human[8e] (n=22)	Human[8f] (n=18)	Human[9g] (n=6)	Human[9h] (n=6)
		**Δ^5-Sterols (µg g^{-1})**				**Sterols (mg day^{-1})**	
Cholesterol	290 ±26‡	1201 ±574‡	nd	nd	nd	79 ±49†	135 ±165†
Stigmasterol	18 ±7	6 ±3	nd	nd	nd	nd	nd
β-Sitosterol	121 ±15	117 ±103	nd	nd	nd	13 ±13	70 ±77
		**5β-Stanols (µg g^{-1})**				**5β-Stanols (mg day^{-1})**	
Coprostanol	3432 ±636	2036 ±577	20600	7400	9300	420 ±138	417 ±164
5β-Stigmastanol	1245 ±263	15 ±4	nd	nd	nd	64 ±14	205 ±93
		**Epi-5β-stanols (µg g^{-1})**				**Epi-5β-stanols (mg day^{-1})**	
Epicoprostanol	52 ±19	0.03 ±0.02	nd	nd	nd	nd	nd
Epi-5β-stigmastanol	0 ±0	nd	nd	nd	nd	nd	nd
		**5α-Stanols (µg g^{-1})**				**5α-Stanols (mg day^{-1})**	
5α-Cholestanol	70 ±15	62 ±38	nd	nd	nd	nd	nd
5α-Stigmastanol	60 ±4	nd	nd	nd	nd	nd	nd
		**Stanones (µg g^{-1})**				**Stanones (mg day^{-1})**	
Coprostanone	29 ±6	nd	nd	nd	nd	27 ±10	63 ±32
Cholestanone	nd	nd	nd	nd	nd	nd	nd
			**Ratio**				
No. I	0.98 ✓	0.97 ✓	-	-	-	-	-
No. II	0.95 ✓	-	-	-	-	-	-
No. III	0.98 ✓	0.97 ✓	-	-	-	-	-
No. IV	-	-	-	-	-	-	-
No. V	73% ✓ (65-81%)	99% ✓ (99-10)	-	-	-	87% ✓ (78-92)	67% ✗ (46-84)
No. VI	0.02 ✓ (0.01-0.03)	-	-	-	-	-	-

nd = not determined; † mean ± standard deviation; ‡ mean ± standard error

We only considered studies, in which steroid contents were presented and quantified (not considered: Bull et al., 1999; Evershed et al., 1997; Jardé et al., 2007; Standley et al., 2000)

Cited studies: 1: Leeming et al., 1996; 3: Shah et al., 2007 (µg g^{-1} wet weight basis); 8: Reddy et al., 1998 (only women); 9: Eneroth et al., 1964 (only men)

3: Results from Shah et al. (2007) presented here but excluded from further discussion due to extremely small 5β-stigmastanol contents compared to all other studies

Diet of the studied humans: x = no information provided; d = Caucasian omnivores; e = Indian vegetarians; f = Caucasian vegetarians; g = dietary fat is butter; h = dietary fat is corn oil

Ratios for a detection of faecal matter (and reference):

No. I: (coprostanol + epicoprostanol) / (5α-cholestanol + coprostanol + epicoprostanol) (Bull et al., 1999);
No. II: (5β-stigmastanol + epi-5β-stigmastanol) / (5α-stigmastanol + 5β-stigmastanol + epi-5β-stigmastanol) (modified from Bull et al., 1999);
No. III: coprostanol / (5α-cholestanol + coprostanol) (Grimalt et al., 1990);
No. IV: coprostanone / (cholestanone + coprostanone) (Grimalt et al., 1990):

Threshold values (Ratios No. I-IV):
>0.7 faecal input confirmed ✓; 0.3-0.7 faecal input can neither be confirmed nor excluded **+/-**; <0.3 faecal input should be excluded ✗

Ratios for a source identification of faecal matter:

No. V: coprostanol / (coprostanol + 5β-stigmastanol) x 100% (Leeming et al., 1997)
No. VI: epi-5β-stigmastanol / 5β-stigmastanol + epicoprostanol / coprostanol

Threshold values (Ratios No. V-VIII):
No. V: < 38% faeces of herbivores; > 73% human faeces (Leeming et al., 1997)
No. VI: > 1.2 horse faeces; < 0.8 no horse faeces

✓ source identification was possible; ✗ source identification was not possible.
