# Supplementary material for: Steroid Biomarkers Revisited – Improved Source Identification of Faecal Remains in Archaeological Soil Material
Source: PLoS One. 2017 Jan 6;12(1):e0164882. doi: 10.1371/journal.pone.0164882 (PMC5217961; doi:10.1371/journal.pone.0164882)
Supplement: S9 Table — (PDF) [file pone.0164882.s035.pdf]

S9 Table. Bile acid contents of omnivore and herbivore faeces (own data and data from literature)

**Steroid (trivial name)**	**Horse**[4x] (n=26)	**Cow**[5b] (n=1)	**Cow**[4x] (n=24)	**Chicken**[4x] (n=17)	**Pig**[4x] (n=26)	**Human**[8d] (n=18)	**Human**[8e] (n=22)	**Human**[8f] (n=18)	**Human**[10x] (n=6)	**Human**[10x] (n=6)
Isodeoxycholic acid (IDCA)	nd	0	nd	nd	nd	nd	nd	nd	500 ±50§	400 ±100§
Isolithocholic acid (ILCA)	nd	2.4 ±0.1†	nd	nd	nd	nd	nd	nd	nd	nd
Lithocholic acid (LCA)	660 ±71‡	25 ±0.4	430 ±36‡	203 ±27‡	6337 ±1661‡	2900 ±700†	1720 ±500†	1630 ±400†	1800 ±100	1200 ±300§
Deoxycholic acid (DCA)	854 ±110	171 ±21	2888 ±444	96 ±12	129 ±17	3210 ±900	2540 ±600	2070 ±900	3500 ±200	2900 ±400
Chenodoxycholic acid (CDCA)	518 ±50	0	0	6638 ±926	1507 ±204	190 ±400	400 ±350	60 ±80	uql	uql
Hyodeoxycholic acid (HDCA)	0	0	0	0	30501 ±4153	nd	nd	nd	nd	nd
Ursodeoxycholic acid (UDCA)	0	0	0	560 ±126	532 ±108	nd	nd	nd	uql	uql
					**Ratio**					
DCA / LCA	1.3 (1.0-1.6)	6.9 (5.9-7.9)	6.7 (5.2-8.5)	0.5 (0.4-0.7)	0.02 (0.01-0.03)	1.1 (0.6-1.9)	1.5 (0.9-2.5)	1.3 (0.6-2.4)	1.9 (1.7-2.2)	2.4 (1.7-3.7)
DCA / CDCA	1.6 (1.3-2.1)	-	-	0.01 (0.01-0.02)	0.09 (0.07-0.11)	17	6.4 (3-63)	35	-	-
CDCA / LCA	0.8 (0.6-1.0)	-	-	32 (25-43)	0.2 (0.2-0.4)	0.07 (0.00-0.3)	0.2 (0.02-0.6)	0.04 (0-0.1)	-	-

nd = not determined; † mean ± standard deviation; ‡ mean ± standard error; § mean ±standard error or standard deviation (no information provided)

Cited studies: 4: Tyagi et al., 2007; 5: Red cattle (own data, composite sample of n=5); 8: Reddy et al., 1998 (only women); Batta et al., 2002 (use of different derivatization agents)

Diet: x = no information provided; b = silage (+ concentrates or + grass); d = Caucasian omnivores; e = Indian vegetarians; f = Caucasian vegetarians
