# Supplementary material for: Steroid Biomarkers Revisited – Improved Source Identification of Faecal Remains in Archaeological Soil Material
Source: PLoS One. 2017 Jan 6;12(1):e0164882. doi: 10.1371/journal.pone.0164882 (PMC5217961; doi:10.1371/journal.pone.0164882)
Supplement: S10 Table — (PDF) [file pone.0164882.s036.pdf]

Supporting Information

"Steroid Biomarkers Revisited – Improved Source Identification of Faecal Remains in Archaeological Soil Material"

S10 Table. Further steroid ratios for source identification of a faecal input applied on steroid contents of faecal samples from this study.

No.	Ratio†	Heck cattle	Sheep	Goats	Horses	Donkey	Geese	Pigs	Humans	Ref.
I	(coprostanol + epicoprostanol) / (5β-stigmastanol + epi-5β-stigmastanol)	0.11 ✓	0.16 ✓	0.07 ✓	0.11 ✓	0.15 ✓	0.20 ✓	0.51 ✗	2.22 ✓	Shillito et al., 2011
II	(coprostanol + 5β-stigmastanol) / (cholesterol + β-sitosterol)	4.6 ✗	4.7 ✗	1.2 ✗	1.7 ✗	3.3 ✗	0.4	8.7	2.2 ✗	Leeming et al., 1996
III	epicoprostanol / (coprostanol + 5α-cholestanol)	0.10 ✗	0.10	0.07	1.04 ✓	0.17	0.05	0.05	0.012 ✗	Standley et al., 2000

All ratios were calculated from the means of n = 3 laboratory replicates, except for the pigs (ratios were calculated from the means of the faecal steroid contents of Turopolje and Mangaliza pigs, n = 2 real replicates with each n = 3 laboratory replicates), range in parentheses.

† Threshold values and interpretation:

No. I: > 1 = omnivore (i.e. human or pig)
No. II: 1 = cows and horses, 1-3 = sheep
No. III: < 0.01 human faeces; > 0.1 cattle and horses

✓ source identification was possible
✗ source identification was not possible
