# Supplementary material for: Steroid Biomarkers Revisited – Improved Source Identification of Faecal Remains in Archaeological Soil Material
Source: PLoS One. 2017 Jan 6;12(1):e0164882. doi: 10.1371/journal.pone.0164882 (PMC5217961; doi:10.1371/journal.pone.0164882)
Supplement: S11 Table — (PDF) [file pone.0164882.s037.pdf]

Supporting Information

“Steroid Biomarkers Revisited – Improved Source Identification of Faecal Remains in Archaeological Soil Material”

S11 Table. Sterol, stanol, and stanone contents of archaeological soil samples.

**Steroid group**	**Δ^5-Sterols**			**5β-stanols**		**Epi-5β-stanols**		**5α-stanols**		**5β-stanone**	**5α-stanone**	**Σ sterols, stanols, stanones**
**Sample**	**Cholesterol**	**β-Sitosterol**	**Stigma-sterol**	**Copro-stanol**	**5β-Stigma-stanol**	**Epicopro-stanol**	**Epi-5β-stigmastanol**	**5α-Chole-stanol**	**5α-Stigma-stanol**	**Copro-stanone**	**Chole-stanone**	
						(µg kg^{-1} soil)						(µg kg^{-1} soil)
**Dormagen**												
Cesspit	26.0 ±0.9	44.7 ±2.2	16.2 ±1.4	22.4 ±0.4	26.5 ±1.7	5.0 ±0.3	9.6 ±1.2	20.7 ±0.7	60.7 ±4.4	4.0 ±0.2	11.4 ±0.3	247
Stable drain (brown filling)[a]	58.0	86.7	0.0	10.0	24.8	7.2	25.3	36.4	94.1	5.1	0	348
Stable drain (green filling)	36.5 ±1.8	25.3 ±0.8	5.6 ±0.3	4.1 ±1.7	6.3 ±4.1	2.9 ±0.6	5.0 ±1.4	20.0 ±2.8	65.3 ±4.4	2.7 ±0.8	0 ±0	174
Stable area	2.1 ±2.0	12.4 ±9.4	2.2 ±1.4	uql	3.6 ±1.7	0 ±0.0	uql	uql	29.6 ±27.0	0 ±0	5.5 ±4.9	55
Control	7.4 ±5.8	40.4 ±5.9	16.5 ±2.6	uql	6.8 ±1.0	uql	2.2 ±0.2	2.0 ±1.5	56.4 ±7.7	0 ±0	3.9 ±3.6	136
**Inden**												
Sewer ditch (70cm)[a]	62.5	42.1	7.2	13.6	34.5	2.6	10.4	21.2	75.7	uql	31.4	304
Sewer ditch (80cm)	109 ±49	101 ±13	8.5 ±12.0	20.3 ±0.2	91.6 ±26.4	14.4 ±1.7	64.6 ±6.3	80.7 ±33.2	126 ±6.7	0 ±0	12.2 ±17.3	631
Control*	21.4	7.9	0	0	4.6	0	4.1	0	50.0	0	16.1	104
**Düren-Arnoldsweiler**												
LBK well[a]	6.8	60.3	8.1	14.6	21.8	2.4	5.5	2.1	61.4	0	62.0	245
Bronze Age well[a]	62.7	376	79.9	25.1	184	52.8	147	33.7	242	0	16.3	1219
Iron Age well[a]	215	1468	188	144	197	50.5	136	337	1533	0	6.5	4275

All values are means ± standard deviation; [a] only one replicate considered (due to low recoveries for the other replicates);

uql = under quantification limit
