# Supplementary material for: Steroid Biomarkers Revisited – Improved Source Identification of Faecal Remains in Archaeological Soil Material
Source: PLoS One. 2017 Jan 6;12(1):e0164882. doi: 10.1371/journal.pone.0164882 (PMC5217961; doi:10.1371/journal.pone.0164882)
Supplement: S12 Table — (PDF) [file pone.0164882.s038.pdf]

Supporting Information

“Steroid Biomarkers Revisited – Improved Source Identification of Faecal Remains in Archaeological Soil Material”

S12 Table. Bile acid contents of archaeological soil samples.

Steroid group	Bile Acids						1st internal Std[b]	Σ Bile Acids
Sample	CDCA	DCA	HDCA	ILCA	LCA	UDCA	IDCA	
	(µg kg^{-1} soil)							(µg kg^{-1} soil)
**Dormagen**								
Cesspit[a]	93.4	769	316	uql	69.5	13.7	0	1262
Stable drain (brown filling)	81.8 ±1.8	450 ±57	104 ±15	uql	88.6 ±0.4	6.2 ±0.2	0	780
Stable drain (green filling)	20.8 ±3.9	192 ±29	89.5 ±56.8	0 ±0	20.7 ±7.9	uql	0	326
Stable area	23.4 ±1.2	213 ±40	41.4 ±20.3	0 ±0	31.6 ±2.5	uql	0	312
Control	12.6 ±1.8	119 ±11	46.7 ±4.9	0 ±0	13.3 ±0.8	0 ±0	0	191
**Inden**								
Sewer ditch (70cm)	26.2 ±7.7	291 ±83	390 ±15	uql	28.0 ±10.2	13.1 ±0.7	0	749
Sewer ditch (80cm)	52.8 ±30.2	235 ±152	118 ±63	5.1 ±2.3	139 ±31	9.1 ±5.7	0	560
Control[a]	10.1	32.2	26.4	0	2.5	0	0	71
**Düren-Arnoldsweiler**								
LBK well	18.6 ±0	701 ±97	1021 ±216	13.9 ±5.7	118 ±22.4	7.9 ±1.8	0	1879
Bronze Age well	21.4 ±3.1	513 ±26	411 ±189	uql	36.5 ±4.8	0 ±0	0	985
Iron Age well	6.4 ±0.9	1306 ±39	237 ±114	uql	208 ±55	0 ±0	0	1758

All values are means ± standard deviation; [a] only one replicate considered (due to low recoveries for the other replicates); [b]quantification by standard addition to the matrix sample;

uql = under quantification limit;

CDCA = chenodeoxycholic acid, DCA = deoxycholic acid, HDCA = hyodeoxycholic acid, ILCA = isolithocholic acid, LCA = lithocholic acid, UDCA = ursodeoxycholic acid, IDCA = isodeoxycholic acid
