# Supplementary material for: Steroid Biomarkers Revisited – Improved Source Identification of Faecal Remains in Archaeological Soil Material
Source: PLoS One. 2017 Jan 6;12(1):e0164882. doi: 10.1371/journal.pone.0164882 (PMC5217961; doi:10.1371/journal.pone.0164882)
Supplement: S1 Text — (PDF) [file pone.0164882.s039.pdf]

Supporting Information

“Steroid Biomarkers Revisited – Improved Source Identification of Faecal Remains in Archaeological Soil Material”

S1 Text. Further Information on archaeological soil samples (site Dormagen)

The Roman fort *Durnomagus* (1st-4th century AD) stationed from the 2nd to the 3rd century AD a 450 men strong cavalry and belonged to the Roman border defence system *limes*, separating *Germania* in the east from the Roman province *Germania inferior* in the west (Müller et al., 1979; Grohmann, 2009). Its remains are located under the present town centre of Dormagen and it has been archaeologically investigated since the 1960s. The samples from this study were taken at an excavation in the years 2007-2008 (Müller et al., 1979; Grohmann, 2009). During this excavation the north-western part of the fort was exposed containing inter alia a combination building with soldier barracks and stable boxes (Fig. S3). Each of the 3.3 x 4m large stable boxes comprised a stable drain in its center, containing a brown filling inside and a green filling at the outsides. Similar features - that had been exposed in earlier excavations - were described as drains of 3m length, 0.6-0.9m width and a trapezoidal cross section with a rounded bottom in 0.6m under the ancient Roman ground surface. All of them showed a greyish green discolouration of the originally yellowish brown soil by reduction processes, at some places even with a penetration depth of 0.5m (Müller et al., 1979; Fig. S1B, S2). Plant remains found in the course of the first excavations of the stable boxes as well as archaeological artefacts - like bridle parts - exposed during later excavations, indicated that the boxes had belonged to horse stables (Müller et al., 1979). The area of the barracks was situated in southern direction, in close proximity to the horse stables. In this area a supposed cesspit was found (Fig. S1A, S3; Grohmann, 2009).
