# Supplementary material for: Steroid Biomarkers Revisited – Improved Source Identification of Faecal Remains in Archaeological Soil Material
Source: PLoS One. 2017 Jan 6;12(1):e0164882. doi: 10.1371/journal.pone.0164882 (PMC5217961; doi:10.1371/journal.pone.0164882)
Supplement: S2 Text — (PDF) [file pone.0164882.s040.pdf]

S2 Text. Further information on archaeological soil samples (site Düren-Arnoldsweiler)

At the site Düren-Arnoldsweiler samples from one well with a box-shaped wooden lining dating to the Linearbandkeramik (Linear Pottery culture, LBK, c. 5300 – 5000 BC), from a tree trunk well from the Bronze Age or Urnfield Period and from a water hole with wickerwork revetment from the Early to Middle Iron Age (c. 544-389 BC, according to AMS radiocarbon dating) had been taken (Fig. S5; Frank, 2014; Husmann and Cziesla, 2014; Jürgens, 2014). According to pollen analyses, showing large values of elm (*Ulmus spp.*) and mugwort (*Artemisia vulgaris*) pollen, the LBK well had been situated in a Neolithic settlement. For the tree trunk well AMS radiocarbon dating indicated a filling of the well at about 1440 ± 35 BC (Bronze Age), whereas pollen analyses, revealing a large input of Linden (*Tilia spp.*) and the absence of beech (*Fagus spp.*) pollen, dated the well to the Urnfield Period (c. 1200 – 700 BC). Large pollen values of ruderal species indicated for the Iron Age water hole the proximity to a settlement. Hence, pollen analyses could reveal for the LBK well and for the Iron Age water hole an on-site and for the tree trunk well from the Bronze Age / Urnfield Period an off-site use. All revetments as well as the organic fillings of the excavated wells and the water hole were well conserved due to their constant position below the groundwater table (Gerlach et al., 2011). This sample set did not contain any control samples.
