# Supplementary material for: Steroid Biomarkers Revisited – Improved Source Identification of Faecal Remains in Archaeological Soil Material
Source: PLoS One. 2017 Jan 6;12(1):e0164882. doi: 10.1371/journal.pone.0164882 (PMC5217961; doi:10.1371/journal.pone.0164882)
Supplement: S3 Text — (PDF) [file pone.0164882.s041.pdf]

S3 Text. Archaeological and historical context for the complete study site region

For the Lower Rhine Basin the **Neolithic Revolution** began at 5300 BC (Linearbandkeramik). The first farmers mainly settled in the loess landscape with its soils providing near-optimal cropping conditions (Lüning, 2000, Zimmermann et al., 2006). For that time the livestock of the Linearbandkeramik culture in Central Europe consisted of cattle, pigs, sheep, and goats (Benecke, 1994a; Lüning, 1997). For the Linearbandkeramik culture the use of goats was mainly concentrated on the mountainous regions of Southeast and Southwest Europe and the Alps (Benecke, 1994b), nevertheless, in the studied area also the presence of another culture - called La Hoguette - cannot be excluded (Zimmermann et al., 2006). The La Hoguette people were pastoralists, keeping sheep and goats, but also lived as hunter-gatherers of possibly local ancestry with cultural links to southern France (Gronenborn, 2007). Hence, all above listed livestock animals, typical for the Early Neolithic, could have been present on the studied sites, but no horses, geese or chicken, as they all were domesticated later (horses from the 4th millennium BC, geese and chicken from the early Iron Age on; Benecke, 1994a). For the Loess region, however, bone remains from the Neolithic are hardly found due to decalcification processes (Eckmeier et al., 2014), i.e., archaeozoological evidence for pigs, cattle, sheep and goats is lacking for such study sites (Kooijmans, 1993). Livestock mainly lived from forest pasture, but during winter time the animals were kept in the villages and fed with collected fodder (i.e. leafs and branches; Lüning, 1997). It is thus, that the idea of a – at least temporarily - presence of pigs in settlements in the Early Neolithic Period is supported by different authors. They state a wood pasture of large herds under the eye of a swineherd but also a winter feeding of pigs in the settlements as well as a penning of the sows during rotting season in order to avoid a cross-breeding of house pigs with the wild boar (Clutton-Brock, 1981; Lüning, 1997). Nevertheless, a crossbreeding of house pigs and the wild boar sometimes occurred intentionally (Lüning, 2000).

For the **Bronze Age** livestock inventory increased with the spreading of horse breeding (Benecke, 1994a), with cattle being the predominating animal (60-80%) followed by pigs and sheep and goats (Arnoldussen and Fokkens, 2008). The feeding of animals occurred for pigs still by forest pasture but also by (penned) grassland for horses and cattle (Clutton-Brock, 1981; Lüning, 1997; Arnoldussen and Fokkens, 2008). In the settlements animals could be kept in the houses, in a byre next to the house or penned nearby (Fokkens, 1998; Fokkens and Arnoldussen, 2008).

In **Iron Age** settlements cattle mostly dominated livestock inventory sometimes in equal proportion with but often followed by pigs, whereas sheep/goats, horses and dogs occurred (in the inland) only in minor proportions (Simons, 1993; Benecke, 1994b; Jockenhövel, 1997). New in livestock inventory was the occurrence of chicken and geese that firstly appeared in Central Europe during the Hallstatt and Latène Period (Benecke, 1994b; Döhle, 2006; with geese representing another herbivore livestock; Moore, 1998).

Both samples dating to the **Roman Age** (i.e. the Roman fort in Dormagen and the sewer ditch in Inden,) are from sites belonging to the so called Roman province *Germania inferior* (c. 83- 400; Bechert, 2007). In the course of the Roman occupation an intensification of the agricultural production was established, i.e. the formation of more farms practicing a market-oriented agriculture with mixed farming systems. This agricultural intensification had to satisfy the large demand for food (cereals, fruits, vegetables, eggs, meat, etc.) and fodder for soldiers, their impedimenta – including draught, pack, and riding animals- and the growing towns (Lüning, 1997; Peters, 1998; Bechert, 2007). This lead to a change in the composition of livestock with a dominance of cattle - used for working (ploughing, draught and pack animals) as well as for meat and leather supply – and an increase in the number of goats (used especially for leather goods) compared to sheep (Peters, 1998). Hence, the composition of livestock in the province *Germania inferior* during the Roman Period was c. 7:2:1 for cattle, pig and sheep/goat (Lüning, 1997; Peters, 1998). Additionally, there was an increase in the breeding of horses to satisfy the great demand of the Roman army, especially in the beginning of the occupation (Peters, 1998). Also for that time, a manual of Roman military institutions (Epitoma rei militaris by Vegetius) recommended the keeping of pigs inside the forts in order to have meat available in the case of siege (Peters, 1998).
