# Supplementary material for: Steroid Biomarkers Revisited – Improved Source Identification of Faecal Remains in Archaeological Soil Material
Source: PLoS One. 2017 Jan 6;12(1):e0164882. doi: 10.1371/journal.pone.0164882 (PMC5217961; doi:10.1371/journal.pone.0164882)
Supplement: S4 Text — (PDF) [file pone.0164882.s042.pdf]

S4 Text. References (Supporting Information)

ARCHE AUSTRIA - Verein zur Erhaltung seltener Nutztierrassen. Turupolje; 2014. Available: www.archepedia.at. Accessed 22 January 2014.

Arnoldussen S, Fokkens H. Bronze Age settlements in the Low Countries: an overview. In: Arnoldussen S, Fokkens H, editors. Bronze Age settlements in the Low Countries; 2008. pp. 17–40.

Batta AK, Salen G, Batta P, Tint SG, Alberts DS, Earnest DL. Simultaneous quantitation of fatty acids, sterols and bile acids in human stool by capillary gas–liquid chromatography. J. Chromatogr. B. 2002; 775(2):153–161.

Bechert T. Germania inferior. Eine Provinz an der Nordgrenze des Römischen Reiches. Mainz am Rhein: Von Zabern; 2007

Becker T. Neue Erkenntnisse zum römischen Auxiliarkastell Dormagen. Archäologie im Rheinland. 2007: 110–112.

Benecke N. Archäozoologische Studien zur Entwicklung der Haustierhaltung. In Mitteleuropa und Südskandinavien von den Anfängen bis zum ausgehenden Mittelalter. Berlin: Akademie Verlag; 1994a.

Benecke N. Der Mensch und seine Haustiere. Die Geschichte einer jahrtausendealten Beziehung. Stuttgart: Theiss; 1994b.

Bull ID, Simpson IA, van Bergen PF, Evershed RP. Muck 'n' molecules: organic geochemical methods for detecting ancient manuring. Antiquity. 1999; 73:86–96

Clutton-Brock J. Domesticated Animals from early times. London: British Museum (Natural History); 1981.

Derrien M, Jarde E, Gruau G, Pierson-Wickmann A. Extreme variability of steroid profiles in cow feces and pig slurries at the regional scale: implications for the use of steroids to specify fecal pollution sources in waters. J. Agric. Food Chem. 2011; 59(13):7294–7302.

Döhle H. Tierknochen aus eisenzeitlichen Siedlungen in Mitteldeutschland. In: Teegen W, Beck H, Müller R, editors. Studien zur Lebenswelt der Eisenzeit. Festschrift für Rosemarie Müller. Berlin: de Gruyter; 2006. pp. 565–590.

Eckmeier E, Altemeier T, Gerlach R. Auswirkungen geochemischer Eigenschaften von Böden auf die Knochenerhaltung in Arnoldsweiler. In: Cziesla E, Ibeling T, editors. Autobahn 4. Fundplatz der Extraklasse. Archäologie unter der neuen Bundesautobahn bei Arnoldsweiler. Langenweißbach: Verlag Beier & Beran. Archäologische Fachliteratur; 2014. pp. 151–154.

Eneroth PK, Hellstrom K, Rhyage R. Identification and quantification of neutral fecal steroids by gas-liquid chromatography and mass-spectrometry: studies of human excretion during two dietary regimes. J. of Lipid Res. 1964; 5:245–262

Evershed RP, Bethell PH, Reynolds PJ, Walsh NJ. 5β-Stigmastanol and related 5β-stanols as biomarkers of manuring: analysis of modern experimental material and assessment of the archaeological potential. J. Archaeol. Sci. 1997; 24: 485–495.

Fokkens H. Cattle and martiality. Changing relations between man and landscape in the Late Neolithic and the Bronze Age. Settlement and Landscape. Proceedings of a conference in Aarhus, Denmark. 1998. 1998: 35–43.

Fokkens H, Arnoldussen S. Towards new models. In: Arnoldussen S, Fokkens H, editors. Bronze Age settlements in the Low Countries; 2008.

Frank T. Die dendrochronologische Untersuchung des hölzernen Brunnenkastens. In: Cziesla E, Ibeling T, editors. Autobahn 4. Fundplatz der Extraklasse. Archäologie unter der neuen Bundesautobahn bei Arnoldsweiler. Langenweißbach: Verlag Beier & Beran. Archäologische Fachliteratur; 2014. pp. 119–124.

Gerlach R, Hilgers A. Grubenfüllungen als archäologische Quelle. In: Bork H, Meller H, Gerlach R, editors. Umweltarchäologie-Naturkatastrophen und Umweltwandel im archäologischen Befund: Tagungen des Landesmuseums für Vorgeschichte Halle (Saale); 2011. pp. 27–36.

Gerlach R, Meurers-Balke J, Kalis AJ, Fischer P, Hilgers A, Protze J. Erste Ergebnisse zur Umweltrekonstruktion in Düren-Arnolsweiler. Archäologie im Rheinland. 2011; 2010: 65–68.

Gesellschaft zur Erhaltung alter und gefährdeter Haustierrassen e. V. (GEH). Deutsche Legegänse; 2014. Available: www.g-e-h.de. Accessed 22 January 2014.

Gill FL, Dewhurst RJ, Dungait JA, Evershed RP, Ives L, Li C, et al. Archaeol – a biomarker for foregut fermentation in modern and ancient herbivorous mammals? Org. Geochem. 2010; 41(5):467–472.

Grimalt JO, Fernández P, Bayona JM, Albalgés J. Assessment of fecal sterols and ketones as indicators of urban sewage inputs to coastal waters. Environ Sci Technol. 1990; 24(3):357–363

Grohmann I. Die Kastellgrabung in Dormagen. Archäologie im Rheinland. 2009; 2008: 88–90.

Gronenborn D. Beyon the models: "Neolithisation" in Central Europe. 2007.

Grunenfelder H, Gugic G, Punz F. Saving the Turopolje pig in Croatia. Anim Genet Resour. 1994; 14: 27–32.

Husmann H, Cziesla E. Bandkeramische Häuser, Brunnen und ein Erdwerk. In: Cziesla E, Ibeling T, editors. Autobahn 4. Fundplatz der Extraklasse. Archäologie unter der neuen Bundesautobahn bei Arnoldsweiler. Langenweißbach: Verlag Beier & Beran. Archäologische Fachliteratur; 2014. pp. 71–118.

Isobe KO, Tarao M, Zakaria MP, Chiem NH, Le Minh Y, Takada H. Quantitative application of fecal sterols using gas chromatography−mass spectrometry to investigate fecal pollution in tropical waters. Western Malaysia and Mekong Delta, Vietnam. Environ Sci Technol. 2002; 36: 4497–4507.

Jardé E, Gruau G, Mansuy-Huault L, Peu P, Martinez J. Using sterols to detect pig slurry contribution to soil organic matter. Water Air Soil Pollut. 2007; 178: 169–178.

Jockenhövel A. Agrargeschichte der Bronzezeit und vorrömischen Eisenzeit (von 2200 v. Chr. bis Christi Geburt). In: Lüning J, Jockenhövel A, Bender H, Capelle T, editors. Deutsche Agrargeschichte, Vor- und Frühgeschichte. Stuttgart: Ulmer; 1997. pp. 141–227.

Jürgens S. Metallzeitliche Funde und Befunde. In: Cziesla E, Ibeling T, editors. Autobahn 4. Fundplatz der Extraklasse. Archäologie unter der neuen Bundesautobahn bei Arnoldsweiler. Langenweißbach: Verlag Beier & Beran. Archäologische Fachliteratur; 2014. pp. 225–272.

Kooijmans LPL. The Mesolithic/Neolithic transformation in the Lower Rhine Basin. In: Bogucki P, editor. Case studies in European prehistory: Taylor & Francis; 1993.

Leeming R, Ball A, Ashbolt N, Nichols P. Using faecal sterols from humans and animals to distinguish faecal pollution in receiving waters. Water Res. 1996; 30(12):2893–2900

Leeming R, Latham V, Rayner M, Nichols P. Detecting and distinguishing sources of sewage pollution in Australian inland and coastal waters and sediments. In: Eganhouse Robert P (ed) Molecular markers in environmental geochemistry. American Chemical Society, Washington, DC; 1997. pp. 306–319.

Lüning J. Anfänge und frühe Entwicklung der Landwirtschaft im Neolithikum (5500–2200 v. Chr.). In: Lüning J, Jockenhövel A, Bender H, Capelle T, editors. Deutsche Agrargeschichte, Vor- und Frühgeschichte. Stuttgart: Ulmer; 1997. pp. 15–139.

Lüning J. Steinzeitliche Bauern in Deutschland. Die Landwirtschaft im Neolithikum. Bonn: Habelt; 2000.

Moore, SJ. The gizzard morphology of an avian herbivore: the domestic goose, *Anser anser*. Aust. J. Zool.1998; 46: 345–357.

Müller G, Horn HG, Chantraine H, Zedelius V. DVRNOMAGVS. Das römische Dormagen. Kunst und Altertum am Rhein. Köln: Führer des Rheinischen Landesmuseums Bonn 90. Dormagener Beiträge 8; 1979.

Oroian IG, Petrescu-Mag IV. Mangalitsa breed returns to homeland. Porcine Res. 2014; 4: 19–21.

Peters J. Römische Tierhaltung und Tierzucht. Eine Synthese aus archäozoologischer Untersuchung und schriftlich-bildlicher Überlieferung. Rahden/Westf.: Leidorf; 1998.

Reddy S, Sanders TAB, Owen RW, Thompson MH. Faecal pH, bile acid and sterol concentrations in premenopausal Indian and white vegetarians compared with white omnivores. Br. J. Nutr. 1998; 79: 495–500.

Sambraus HH. Gefährdete Nutztierrassen. Ihre Zuchtgeschichte, Nutzung und Bewahrung. 2nd ed. Stuttgart (Hohenheim): E. Ulmer; 1999.

Sambraus HH. Farbatlas Nutztierrassen. 7th ed. Stuttgart: Ulmer; 2011.

Shah VG, Dunstan RH, Geary PM, Coombes P, Roberts TK, Nagy-Felsobuki E von. Evaluating potential applications of faecal sterols in distinguishing sources of faecal contamination from mixed faecal samples. Water Res. 2007; 41(16):3691–3700.

Shillito L, Bull ID, Matthews W, Almond MJ, Williams JM, Evershed RP. Biomolecular and micromorphological analysis of suspected faecal deposits at Neolithic Çatalhöyük, Turkey. J. Archaeol. Sci. 2011; 38: 1869–1877.

Simons A. Wirtschafts- und Siedlungsweisen in der Bronze- und Eisenzeit des Rheinlandes. In 7000 Jahre bäuerliche Landschaft: Entstehung, Erforschung, Erhaltung. Archaeo-Physica. 1993.

Standley L, Kaplan L, Smith D. Molecular tracers of organic matter sources to surface water resources. Environ Sci Technol. 2000; 34(15):3124–3130

Tyagi P, Edwards DR, Coyne MS. Use of selected chemical markers in combination with a multiple regression model to assess the contribution of domesticated animal sources of fecal pollution in the environment. Chemosphere. 2007; 69(10):1617–1624

Zimmermann A, Meurers-Balke J, Kalis AJ. Das Neolithikum. In: Kunow J, Wegener H, Kunow J, Wegner H, editors. Urgeschichte im Rheinland. Köln: Verlag des Rheinischen Verein für Denkmalpflege und Landschaftsschutz; 2006. pp. 159–202.
